# Urine protein:creatinine ratio vs 24-hour urine protein for proteinuria management: analysis from the phase 3 REFLECT study of lenvatinib vs sorafenib in hepatocellular carcinoma

**DOI:** 10.1038/s41416-019-0506-6

**Published:** 2019-06-28

**Authors:** Thomas R. Jeffry Evans, Masatoshi Kudo, Richard S. Finn, Kwang-Hyub Han, Ann-Lii Cheng, Masafumi Ikeda, Silvija Kraljevic, Min Ren, Corina E. Dutcus, Fabio Piscaglia, Max W. Sung

**Affiliations:** 1 University of Glasgow, Beatson West of Scotland Cancer Centre, Glasgow, UK; 2 Department of Gastroenterology and Hepatology, Kindai University Faculty of Medicine, Osaka, Japan; 3 David Geffen School of Medicine, UCLA Medical Center, Los Angeles, CA USA; 4 Severance Hospital, Yonsei University, Seoul, South Korea; 5 National Taiwan University Hospital and National Taiwan University Cancer Center, Taipei, Taiwan; 6 Department of Hepatobiliary and Pancreatic Oncology, National Cancer Center Hospital East, Kashiwa, Japan; 7 Former employee of Eisai Ltd, Hatfield, UK; 8 Eisai Inc., Woodcliff Lake, NJ USA; 9 Unit of Internal Medicine, University of Bologna, S.Orsola-Malpighi Hospital, Bologna, Italy; 10 Tisch Cancer Institute at Mount Sinai, New York, NY USA

**Keywords:** Cancer, Cancer

## Abstract

**Background:**

Proteinuria monitoring is required in patients receiving lenvatinib, however, current methodology involves burdensome overnight urine collection.

**Methods:**

To determine whether the simpler urine protein:creatinine ratio (UPCR) calculated from spot urine samples could be accurately used for proteinuria monitoring in patients receiving lenvatinib, we evaluated the correlation between UPCR and 24-hour urine protein results from the phase 3 REFLECT study. Paired data (323 tests, 154 patients) were analysed.

**Results:**

Regression analysis showed a statistically significant correlation between UPCR and 24-hour urine protein (*R*^2^: 0.75; *P* < 2 × 10^−16^). A UPCR cut-off value of 2.4 had 96.9% sensitivity, 82.5% specificity for delineating between grade 2 and 3 proteinuria. Using this UPCR cut-off value to determine the need for further testing could reduce the need for 24-hour urine collection in ~74% of patients.

**Conclusion:**

Incorporation of UPCR into the current algorithm for proteinuria management can enable optimisation of lenvatinib treatment, while minimising patient inconvenience.

**Clinical trial registration:**

NCT01761266

## Background

Lenvatinib is a multikinase inhibitor of vascular endothelial growth factor receptors (VEGFR) 1‒3, fibroblast growth factor receptors 1–4, platelet-derived growth factor-alpha, KIT, and RET.^1,2^ Lenvatinib monotherapy is indicated for first-line treatment of unresectable hepatocellular carcinoma (HCC)^3,4^ based on results from the phase 3 REFLECT study, where lenvatinib demonstrated a treatment effect on overall survival with statistical confirmation of noninferiority to sorafenib (13.6 vs 12.3 months, respectively; hazard ratio 0.92; 95% confidence interval 0.79–1.06), along with significant improvements in progression-free survival, time to progression, and objective response rates.^5^ Lenvatinib is also indicated as monotherapy for patients with locally recurrent or metastatic, progressive, radioiodine-refractory differentiated thyroid cancer (RR-DTC), and in combination with everolimus in patients with advanced renal cell carcinoma (RCC) following 1 prior anti-angiogenic therapy.^3^

Proteinuria is a class effect of antiangiogenic agents,^6^ and a well-documented lenvatinib-associated adverse effect.^5,7–9^ Rates of any-grade/grade ≥3 proteinuria observed in lenvatinib-treated patients were 25%/6% in the phase 3 REFLECT study in unresectable HCC,^5^ 31%/10% in the phase 3 SELECT in patients with RR-DTC,^8^ and 31%/19% in a phase 2 study in patients with advanced/metastatic RCC.^7^

Patients receiving lenvatinib are monitored regularly for proteinuria using a urine sample dipstick method. The current standard management requires a 24-hour urine protein test if a dipstick proteinuria result of ≥2+ is detected, with the recommendation that lenvatinib treatment should be withheld if a proteinuria level of ≥2 g/24 h is detected.^3^ This 24-hour urine protein test relies on patient collection of urine overnight, which is burdensome and may be influenced by patient compliance. However, the single (“spot”) urine protein:creatinine ratio (UPCR) is a simple and convenient alternative test that is often used to detect proteinuria associated with certain medical conditions, e.g., chronic kidney disease and diabetes.^10,11^ UPCR is calculated by dividing the level of protein (mg/dl) in a spot urine test by the creatinine level (mg/dl).^12^ This approach was first validated in 1983 by Ginsberg and colleagues.^13^ It is based on the premise that urinary creatinine excretion and the protein excretion rate in the presence of a stable glomerular filtration rate are fairly constant in a given patient. Thus, a simple ratio of the 2 in a single-void urine sample would reflect cumulative protein excretion over a day (because the ratio of 2 stable rates would cancel out the time factor).

To determine whether UPCR could be a useful and more convenient assessment for proteinuria in patients receiving lenvatinib, we evaluated the correlation between proteinuria assessment by UPCR and 24-hour urine protein in patients with HCC from the REFLECT study.

## Materials and methods

### Study design and patients

Details of the REFLECT study have been published previously.^5^ Briefly, REFLECT was an international, randomised, open-label, noninferiority study, which enroled 954 previously untreated patients with unresectable HCC from 154 sites in 20 countries throughout Asia-Pacific, European, and North American regions. Patients were randomised (1:1) to receive lenvatinib (*n* = 478) or sorafenib (*n* = 476).

### End points and assessments

All patients underwent urine dipstick testing at each scheduled safety assessment visit (day 1 and day 15 of the first 2 cycles, then day 1 of each subsequent cycle). Within 72 h of a positive (≥2) proteinuria urine dipstick test during regular safety assessment visits, patients underwent standard-of-care 24-hour urine collection for total protein, performed either at the central or local site laboratories, as well as a UPCR test, performed at the central laboratory. 24-Hour urine collection was used to grade proteinuria according to Common Terminology Criteria for Adverse Events (CTCAE) v4.0 criteria (Supplementary Table 1). Patients with positive urine dipstick test underwent dipstick testing every 2 weeks until results were reduced to 1+ or negative for 3 consecutive months.

### Statistical analyses

Paired data were analysed. Optimal UPCR cut-off values were identified using standard receiver operating characteristic methods to maximise the Youden index/statistics (sensitivity + specificity), with urine data dichotomised by CTCAE proteinuria grades (grade <2 vs ≥2 and grade ≤2 vs 3). The correlation between 24-hour urine protein data (as continuous values) and UPCR was analysed using a regression model of log-transformed data. Also, the optimal UPCR cut-off value determining which patients should undergo further testing with 24-hour urine collection was calculated. Statistical analyses were performed using R statistical software.

## Results

Paired data (323 paired tests) from 154 patients were included in the analysis.

### Cut-off values for UPCR

The optimal cut-off to discriminate grade 1 (1+ proteinuria; urinary protein <1.0 g/24 h) from grade ≥2 (2+ proteinuria; urinary protein >1.0 g/24 h) proteinuria by UPCR was 1.02, (94.0% sensitivity; 72.4% specificity) (Supplementary Fig. 1A). The optimal cut-off to discriminate grade 2 from grade 3 proteinuria by UPCR was 2.43 (96.9% sensitivity; 82.5% specificity) (Supplementary Fig. 1B), the positive likelihood ratio was 5.54, and the negative likelihood ratio was 0.038. For ease of clinical use, a cut-off of 2.4 is proposed.

### Correlation between 24-hour urine protein collection and UPCR

**Fig. 1:**
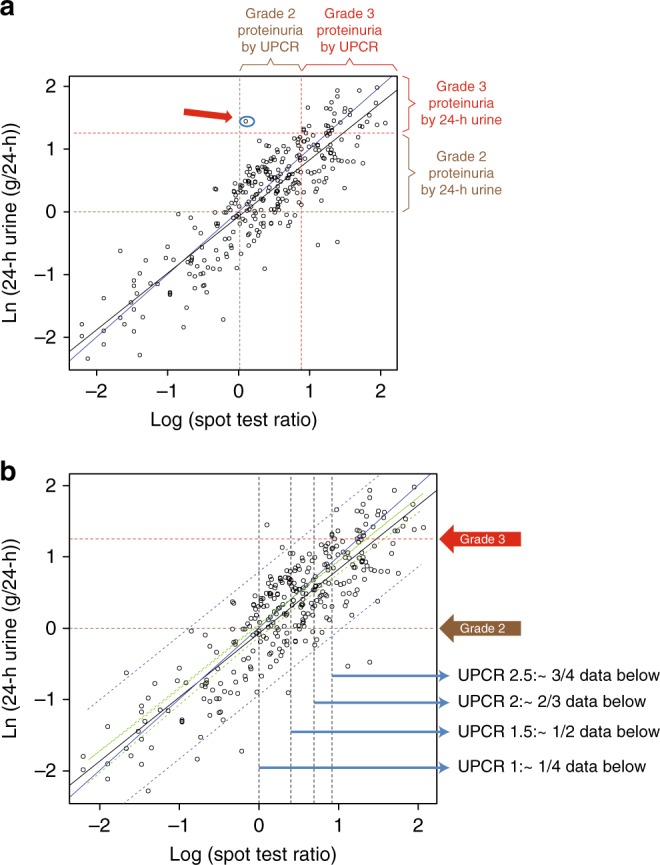
**a** Correlation between 24-hour urine protein collection and UPCR, analysed using a regression model of log-transformed data (323 paired tests). The red arrow indicates a single test that had grade 3 proteinuria based on 24-hour urine collection but not with UPCR based on the optimal UPCR cut-off value of 2.4; **b** proposed UPCR cut-off values for determining whether to perform 24-hour urine collection. Units of measurement are mg/dl for urine protein, and ratio for UPCR. Ln, natural log; UPCR, urine protein:creatinine ratio

After removal of a single outlier from the regression model, regression analysis of UPCR versus 24-hour urine protein collection data showed a statistically significant correlation, with a Pearson correlation coefficient (*R*) of 0.86, an *R*^2^ of 0.75, and slope of 0.9 (*P* < 2 × 10^−16^) (Fig. 1a). Below the grade 3 optimal UPCR cut-off level (2.4), only 1 test out of 239 had grade 3 proteinuria based on 24-hour urine collection but not based on UPCR (Fig. 1a). Using the grade 1 + 2 versus grade 3 proteinuria UPCR cut-off of 2.4 as an optimal cut-off to determine which patients should undergo further testing with 24-hour urine collection, 239/323 pairs of tests were below or equal to this cut-off and only 1 test was missed (0.4%); thus, the need for 24-hour urine collection would be reduced by 74% (Fig. 1b). Also, 84/323 (26%) pairs of tests were above this cut-off, with 53/84 (63%) pairs being grade 1 + 2 proteinuria.

## Discussion

The mechanism underlying VEGFR-inhibitor-associated proteinuria are unclear, but may involve thrombotic microangiopathy or impairment of podocyte function.^14^ However, proteinuria is a manageable adverse event that does not typically lead to clinically meaningful adverse outcomes if it is appropriately monitored and managed.^3,14^ The regression model of the UPCR versus 24-hour urine protein data suggests a strong and statistically significant correlation between the 2 measurements, and using a UPCR cut-off value of 2.4 to delineate between grade 2 and 3 proteinuria and the need for further testing would obviate the need for the more burdensome 24-hour urine collection for an estimated 74% of patients with urine protein dipstick results of ≥2+.

Introduction of UPCR into the guidelines for proteinuria management could be as follows and as described in Supplementary Table 2: urine dipstick testing would be performed as regularly scheduled. A 24-hour urine collection *or* an immediate spot UPCR test would be required in the case of: (1) first occurrence of ≥2+ proteinuria while on lenvatinib; (2) a subsequent increase in severity of urine dipstick proteinuria while on the same dose level; (3) when following a lenvatinib dose reduction, the urine protein dipstick result was ≥2+. In addition, a 24-hour urine collection should be initiated as soon as possible (within 72 h) when UPCR is ≥2.4 to verify the grade of proteinuria. After the proteinuria has improved to a lower grade, lenvatinib may be restarted at a reduced dose. By following these criteria, proteinuria can be safely managed, enabling optimisation of lenvatinib treatment while minimising inconvenience to patients. It should also be noted that, although this analysis was conducted in patients with HCC, this monitoring approach may potentially be useful in other tumour types treated with lenvatinib (e.g. differentiated thyroid cancer or renal cell carcinoma).

## Conclusions

Data from this large study support the use of UPCR for proteinuria monitoring in patients with unresectable HCC who are receiving lenvatinib therapy and have ≥2+ urine protein during routine monitoring, similar to its use in other diseases. Use of this test would alleviate the need for 24-hour urine collection in most cases, therefore reducing patient burden.

## Supplementary information

Supplementary Appendix

IRB/Ethics Committee Information

## Data Availability

The datasets generated during and/or analysed during the current study are on file with Eisai and not publicly available.
