# Correction: Urine protein:creatinine ratio vs 24-hour urine protein for proteinuria management: analysis from the phase 3 REFLECT study of lenvatinib vs sorafenib in hepatocellular carcinoma

**DOI:** 10.1038/s41416-019-0534-2

**Published:** 2019-07-30

**Authors:** Thomas R. Jeffry Evans, Masatoshi Kudo, Richard S. Finn, Kwang-Hyub Han, Ann-Lii Cheng, Masafumi Ikeda, Silvija Kraljevic, Min Ren, Corina E. Dutcus, Fabio Piscaglia, Max W. Sung

**Affiliations:** 1 University of Glasgow, Beatson West of Scotland Cancer Centre, Glasgow, UK; 2 Department of Gastroenterology and Hepatology, Kindai University Faculty of Medicine, Osaka, Japan; 3 David Geffen School of Medicine, UCLA Medical Center, Los Angeles, CA USA; 4 Severance Hospital, Yonsei University, Seoul, South Korea; 5 National Taiwan University Hospital and National Taiwan University Cancer Center, Taipei, Taiwan; 6 Department of Hepatobiliary and Pancreatic Oncology, National Cancer Center Hospital East, Kashiwa, Japan; 7 Former employee of Eisai Ltd, Hatfield, UK; 8 Eisai Inc., Woodcliff Lake, NJ USA; 9 Unit of Internal Medicine, University of Bologna, S.Orsola-Malpighi Hospital, Bologna, Italy; 10 Tisch Cancer Institute at Mount Sinai, New York, NY USA

**Keywords:** Cancer, Cancer

**Correction to:** *British Journal of Cancer;* 10.1038/s41416-019-0506-6; published online 28 June 2019.

This article was originally published under a standard license to Publish, but has now been made available under a CC BY license. The PDF and HTML versions of the paper have been modified accordingly.

