# Supplementary material for: Urine protein:creatinine ratio vs 24-hour urine protein for proteinuria management: analysis from the phase 3 REFLECT study of lenvatinib vs sorafenib in hepatocellular carcinoma
Source: Br J Cancer. 2019 Jun 28;121(3):218–21. doi: 10.1038/s41416-019-0506-6 (PMC6738107; doi:10.1038/s41416-019-0506-6)
Supplement: Supplementary file 1 — Supplementary Appendix [file 41416_2019_506_MOESM1_ESM.doc]

**Supplementary Appendix**

**Supplementary Figure 1.** Optimal urine protein:creatinine ratio cut-off values for (**A**) grade <2 versus grade ≥2 proteinuria, and (**B**) grade 2 versus grade 3 proteinuria.

**Supplementary Table 1.** Common Terminology Criteria for Adverse Events version 4.0 grades for proteinuria.

**Supplementary Table 2.** Proposed Use of UPCR for Proteinuria Management With Lenvatinib in uHCC.

**Supplementary Figure 1.** Optimal urine protein:creatinine ratio cut-off values for (**A**) grade <2 versus grade ≥2 proteinuria, and (**B**) grade 2 versus grade 3 proteinuria.


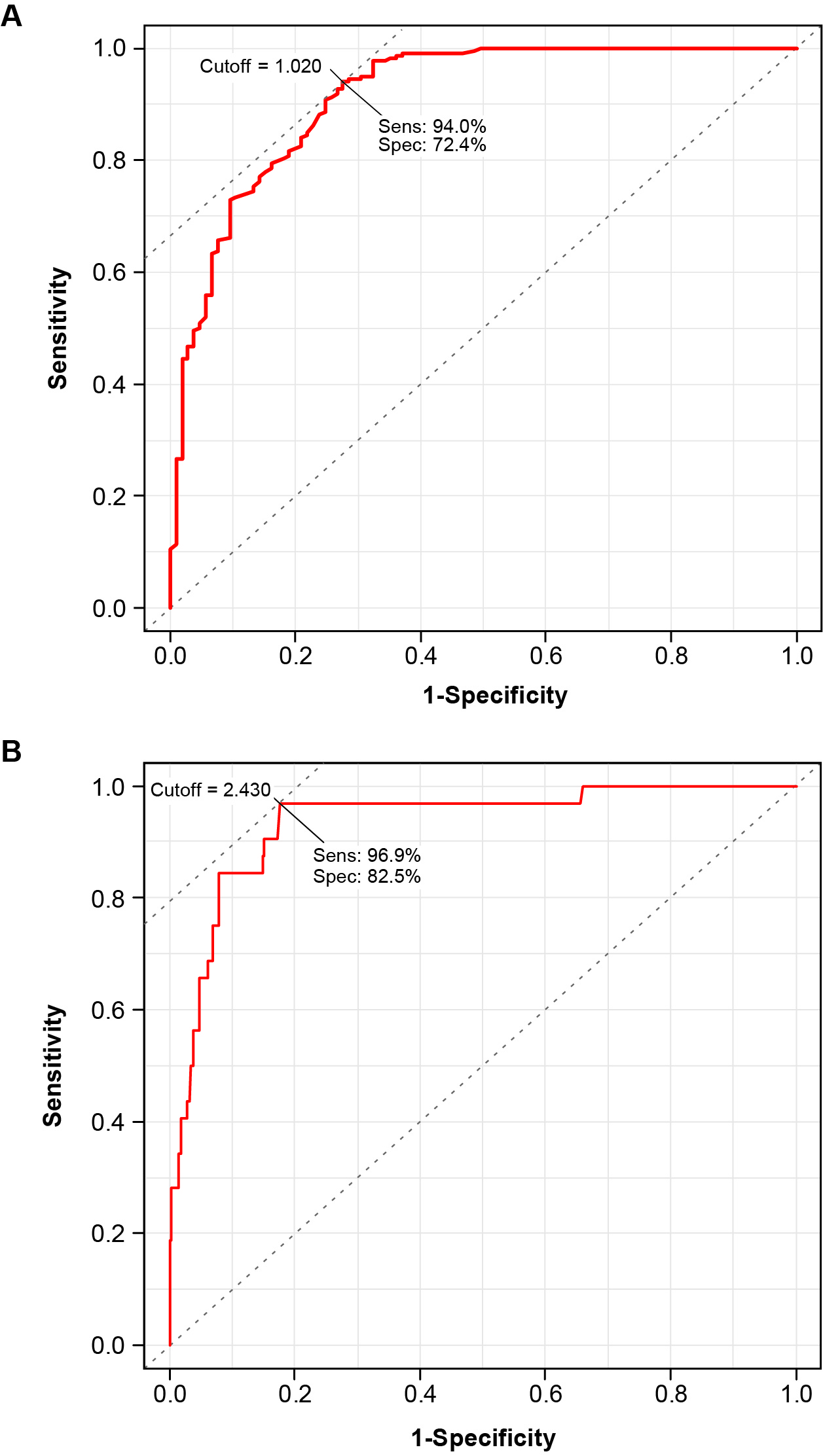


**Supplementary Table 1.** Common Terminology Criteria for Adverse Events version 4.0 grades for proteinuria in adults.

| **Adverse Event** | **Grade** | | |
| --- | --- | --- | --- |
|  | **1** | **2** | **3** |
| Proteinuria | 1+ proteinuria; urinary protein <1.0 g/24 hours | 2+ proteinuria; urinary protein 1.0–3.4 g/24 hours | Urinary protein ≥3.5 g/24 hours |

National Cancer Institute Common Terminology Criteria for Adverse Events version 4.0., published May 28, 2009. <https://evs.nci.nih.gov/ftp1/CTCAE/CTCAE_4.03/Archive/CTCAE_4.0_2009-05-29_QuickReference_8.5x11.pdf>. Accessed 14 January2019.

**Supplementary Table 2:** Proposed Use of UPCR for Proteinuria Management With Lenvatinib in uHCC.

| - Urine dipstick testing would be performed as regularly scheduled |
| --- |
| - A 24-hour urine collection or an immediate spot UPCR test would be required in the case of:   (1) first occurrence of ≥ 2+ proteinuria while on lenvatinib  (2) a subsequent increase in severity of urine dipstick proteinuria while on the same dose level  (3) when following a lenvatinib dose reduction, the urine protein dipstick result was ≥2+ |
| - In addition, a 24-hour urine collection should be initiated as soon as possible (within 72 hours) when UPCR is ≥2.4 to verify the grade of proteinuria |
| - After the proteinuria has improved to a lower grade, lenvatinib may be restarted at a reduced dose |
| - By following these criteria, proteinuria can be safely managed, enabling optimization of lenvatinib treatment while minimizing inconvenience to patients |

UPCR, urine protein:creatinine ratio.
