# Supplementary material for: Urine protein:creatinine ratio vs 24-hour urine protein for proteinuria management: analysis from the phase 3 REFLECT study of lenvatinib vs sorafenib in hepatocellular carcinoma
Source: Br J Cancer. 2019 Jun 28;121(3):218–21. doi: 10.1038/s41416-019-0506-6 (PMC6738107; doi:10.1038/s41416-019-0506-6)
Supplement: Supplementary file 2 — IRB/Ethics Committee Information [file 41416_2019_506_MOESM2_ESM.pdf]

16.1.3 List of IECs or IRBs, Sample Informed Consent Form, and Representative Written Information for Subjects

16.1.3.1 List of IECs or IRBs

The list of IRBs and IECs is provided below.

COUNTRY	SITE	PRINCIPAL INVESTIGATOR	IRB / IEC INFORMATION
United States (USA)	1002	Aiwu Ruth He	MedStar Health Research Institute - Georgetown University Oncology Institutional Review Board<br>3900 Reservoir Road, NW<br>Medical Dental Building, SW 104<br>Washington, DC 20057
United States (USA)	1003	Max W. Sung	Biomedical Research Alliance of New York, LLC / Institutional Review Board<br>1981 Marcus Avenue, Suite 210<br>Lake Success, NY 11042
United States (USA)	1004	Christopher Thomas Hagenstad	Copernicus Group Institutional Review Board<br>One Triangle Drive, Suite 100<br>Durham, NC 27709<br><br>*(Mailing Address:)*<br>P.O. Box 110605<br>Research Triangle Park, NC 27709
United States (USA)	1005	Boone Wilder Goodgame	Copernicus Group Institutional Review Board<br>One Triangle Drive, Suite 100<br>Durham, NC 27709<br><br>*(Mailing Address:)*<br>P.O. Box 110605<br>Research Triangle Park, NC 27709
United States (USA)	1006	Avi David Baron	Western Institutional Review Board<br>1019 39th Avenue SE, Suite 120<br>Puyallup, WA 98374-2115<br><br>*(Previous Address)*<br>3535 Seventh Avenue SW<br>Olympia, WA 98502
United States (USA)	1007	Edward J. Kim	Institutional Review Board<br>University of California – Davis<br>2921 Stockton Boulevard, Suite 1400<br>CTSC Building, Room 1429<br>Sacramento, CA 95817

COUNTRY	SITE	PRINCIPAL INVESTIGATOR	IRB / IEC INFORMATION
United States (USA)	1008	Michael A. Morse	Duke University Health System Institutional Review Board<br>Duke University Medical Center – Box 2712<br>Hock Plaza, Suite 405<br>2424 Erwin Road<br>Durham, NC 27705
United States (USA)	1009	Bradley L. Freilich	Midwest Health System Institutional Review Board<br>903 East 104th Street, 5th Floor<br>Kanas City, MO 64131
United States (USA)	1010	Benjamin R. Tan, Jr.	Human Research Protection Office (IRB)<br>Washington University School of Medicine<br>660 S. Euclid Avenue, Box 8089<br>St. Louis, MO 63110
United States (USA)	1011	Kiran Yalamanchili	St. Joseph Health Institutional Review Board<br>3345 Michelson Drive, Suite 100<br>Irvine, CA 92612
United States (USA)	1012	William Proctor Harris	Western Institutional Review Board<br>1019 39th Avenue SE, Suite 120<br>Puyallup, WA 98374<br><br>*Previous Address :*<br>3535 Seveth Avenue SW<br>Olympia, WA 98502
United States (USA)	1013	Kimberly Ann Brown	Henry Ford Health System Institutional Review Board<br>2799 West Grand Boulevard<br>CFP-Basement, Room 046<br>Detroit, MI 48202
United States (USA)	1014	Paul Joseph Thuluvath	Mercy Medical Center Institutional Review Board<br>345 St. Paul Place<br>Bunting Building, 7th Floor<br>Baltimore, MD 21202
United States (USA)	1015	Richard S. Finn	UCLA Office of the Human Research Protection Program (OHRPP)<br>11000 Kinross Avenue, Suite 211<br>Los Angeles, CA 90095

COUNTRY	SITE	PRINCIPAL INVESTIGATOR	IRB / IEC INFORMATION
United States (USA)	1016	Angel E. Alsina	Copernicus Group Institutional Review Board<br>One Triangle Drive, Suite 100<br>Durham, NC 27709<br><br>*(Mailing Address:)*<br>P.O. Box 110605<br>Research Triangle Park, NC 27709
United States (USA)	1017	Fengming Zhong	Veterans Administration New Jersey Health Care System Human Studies Subcommittee<br>Institutional Review Board<br>385 Tremont Avenue<br>East Orange, NJ 07018
United States (USA)	1018	Kevin Becker	Maimonides Medical Center<br>Institutional Review Board<br>4802 10th Avenue<br>Brooklyn, NY 11219
United States (USA)	1019	Reza Nazemzadeh	Chesapeake Research Review<br>6940 Columbia Gateway Drive, Suite 110<br>Columbia, MD 21046
United States (USA)	1020	Abdul Hai Mansoor	Kaiser Permanente Northwest Institutional Review Board<br>Research Subjects Protection Office<br>3800 N. Interstate Ave.<br>Portland, OR 97227
Japan (JPN)	1201	Kunihiko Tsuji	The Institutional Review Board of Teine Keijinkai Hospital<br>1-12-1-40, Maeda, Teine-ku<br>Sapporo, Hokkaido 006-8555
Japan (JPN)	1202	Yoshiyasu Karino	The Institutional Review Board of Sapporo-Kosei General Hospital<br>8-5, Kita 3-jo Higashi Chuo-ku<br>Sapporo-shi, Hokkaidou
Japan (JPN)	1203	Tatsuya Yamashita	Kanazawa University Hospital Internal Review Board<br>13-1 Takara-machi<br>Kanazawa, Ishikawa 920-8641
Japan (JPN)	1204	Masafumi Ikeda	National Cancer Center Institutional Review Board<br>5-1-1 Tsukiji, Chuo-ku<br>Tokyo 104-0045
Japan (JPN)	1205	Takuji Okusaka	National Cancer Center Institutional Review Board<br>5-1-1 Tsukiji, Chuo-ku<br>Tokyo 104-0045

COUNTRY	SITE	PRINCIPAL INVESTIGATOR	IRB / IEC INFORMATION
Japan (JPN)	1206	Masahiro Kobayashi	The Institutional Review Board of Toranomon Hospital and Toranomon Hospital Kajigaya<br>2-2-2 Toranomon, Minato-ku<br>Tokyo 1058470
Japan (JPN)	1207	Kenji Ikeda	The Institutional Review Board of Toranomon Hospital and Toranomon Hospital Kajigaya<br>2-2-2 Toranomon, Minato-ku<br>Tokyo 1058470
Japan (JPN)	1208	Namiki Izumi	The Institutional Review Board of Musashino Red Cross Hospital<br>1-26-1 kyonan cho, Musashino-city<br>Tokyo 180-8610
Japan (JPN)	1209	Kazushi Numata	The Institutional Review Board of Yokohama City University Medical Center<br>4-57, Urafune-cho, Minami-ku, Yokohama<br>Kanagawa 232-0024
Japan (JPN)	1210	Naoki Sasahira	The Cancer Institute Hospital JFCR Institutional Review Board<br>3-8-31, Ariake, Koto-ku<br>Tokyo 135-8550
Japan (JPN)	1211	Ryosuke Tateishi	The Institutional Review Board of The University of Tokyo Hospital<br>7-3-1 Hongo Bunkyo-Ku<br>Tokyo 113-8655
Japan (JPN)	1212	Yoshitaka Inaba	The Institutional Review Board of Aichi Cancer Center Hospital<br>1-1 Kanokoden, Chikusa-ku, Nagoya-shi<br>Aichi 464-8681
Japan (JPN)	1213	Yoshiyuki Takei	The Institutional Review Board of Mie University Hospital<br>2-174 Edobashi, Tsu<br>Mie 514-8507
Japan (JPN)	1214	Masatoshi Kudo	The Institutional Review Board of Kinki University Hospital<br>377-2, Ohno-Higashi, Osaka-Sayama<br>Osaka 589-8511
Japan (JPN)	1215	Yukio Osaki	The Institutional Review Board of Osaka Red Cross Hospital<br>5-30 Fudegasaki-cho, Tennoji-ku, Osaka-shi<br>Osaka 543-8555
Japan (JPN)	1216	Shinichiro Nakamura	Okayama University Hospital Institutional Review Board<br>2-5-1, shikata-chou, kita-ku, Okayama-shi<br>Okayama 700-8558

COUNTRY	SITE	PRINCIPAL INVESTIGATOR	IRB / IEC INFORMATION
Japan (JPN)	1217	Akira Kato	The Institutional Review board of Shimonoseki Kohsei Hospital\ 3-3-8 Kamishinchi-cho, shimonoseki-city Yamaguchi 750-0061
Japan (JPN)	1218	Seijin Nadano	National Hospital Organization Shikoku Cancer Center Internal Review Board 160, Minamiumemoto-machi-kou Matsuyama-city, Ehime 791-0280
Japan (JPN)	1219	Ryoko Kuromatsu	The Institutional Review Board of Kurume University Hospital 67 Asahi-machi, Kurume-shi Fukuoka 830-0011
Japan (JPN)	1220	Yasunori Kawaguchi	Saga-Ken Medical Center Koseikan Institutional Review Board 400 Nakabaru, Kase-machi, Saga-city Saga 840-8571
Japan (JPN)	1221	Iwata Ozaki	Saga University Hospital Institutional Review Board 5-1-1 Nabeshima, Saga-city Saga 849-8501
Japan (JPN)	1222	Yuko Takami	National Hospital Organization Kyushu medical Center Internal Review Board 1-8-1, Jigyouhama, Chuo-ku Fukuoka 810-8563
Japan (JPN)	1223	Seigo Abiru	National Hospital Organization Nagasaki Medical Center Institutional Review Board 2-1001-1 Kubara, Omura-city Nagasaki 856-8562
Japan (JPN)	1224	Naoto Ikeda	The Institutional Review Board of Hyogo College of Medicine Hospital 1-1, Mukogawa, Nishinomiya Hyogo 663-8501
Japan (JPN)	1225	Hiroshi Aikata	Hiroshima University Hospital Institutional Review Board 1-2-3 Kasumi, Minami-ku, Hiroshima-shi Hiroshima 734-8551
Australia (AUS)	1301	Gary Peter Jeffrey	Sir Charles Gairdner Group Human Research Ethics Committee Level 2, A Block, Hospital Avenue, Nedlands, Western Australia 6009

COUNTRY	SITE	PRINCIPAL INVESTIGATOR	IRB / IEC INFORMATION
Australia (AUS)	1302	Simone I. Strasser	St Vincent’s Hospital (Melbourne) Human Research Ethics Committee-D<br>St Vincent’s Hospital (Melbourne)<br>41 Victoria Parade<br>Fitzroy Victoria, 3065
Australia (AUS)	1303	Jacob George	St Vincent’s Hospital (Melbourne) Human Research Ethics Committee-D<br>St Vincent’s Hospital (Melbourne)<br>41 Victoria Parade<br>Fitzroy, VIC 3065
Australia (AUS)	1304	Stuart Keith Roberts	The Alfred Hospital Ethics Committee<br>Ethics & Research Governance, Ground Floor<br>Linay Pavilion, The Alfred Hospital<br>55 Commercial Road<br>Melbourne, Victoria 3004
Australia (AUS)	1305	Paul V. Desmond	St. Vincent’s Hospital (Melbourne) Human Research Ethics Committee-D<br>St Vincent’s Hospital (Melbourne)<br>41 Victoria Parade<br>Fitzroy, VIC 3065
Australia (AUS)	1306	Lara Rachel Lipton	St. Vincent’s Hospital (Melbourne) Human Research Ethics Committee-D<br>St Vincent’s Hospital (Melbourne)<br>41 Victoria Parade<br>Fitzroy, Victoria 3065
Australia (AUS)	1307	Graeme Alistair Macdonald	St. Vincent’s Hospital (Melbourne) Human Research Ethics Committee-D<br>St Vincent’s Hospital (Melbourne)<br>41 Victoria Parade<br>Fitzroy, VIC 3065
France (FRA)	1401	Eric Assenat	CPP Sud Ouest et Outre Mer III<br>Service Pharmacologie Clinque Bat 1A Hopital Pellegrin<br>Place Amelie Raba Leon<br>Bordeaux 33076 cedex
France (FRA)	1402	Jean-Frederic Blanc	CPP Sud Ouest et Outre Mer III<br>Hopital Pellegrin-service pharmacologie Clinique Bat 1A<br>Place Amelie Raba Leon<br>Bordeaux 33076 cedex

COUNTRY	SITE	PRINCIPAL INVESTIGATOR	IRB / IEC INFORMATION
France (FRA)	1403	Marc Pracht	CPP Sud Ouest et Outre Mer III<br>Hopital Pellegrin-service pharmacologie Clinique Bat 1A<br>Place Amelie Raba Leon<br>Bordeaux 33076 cedex
France (FRA)	1404	Jean-Pierre Bronowicki	CPP Sud Ouest et Outre Mer III<br>Service Pharmacologie Clinque Bat 1A Hopital Pellegrin<br>Place Amelie Raba Leon<br>Bordeaux 33076
France (FRA)	1405	Giuliana Amaddeo	CPP Sud Ouest et Outre Mer III<br>Service Pharmacologie Clinque Bat 1A Hopital Pellegrin<br>Place Amelie Raba Leon<br>Bordeaux 33076 Cedex
France (FRA)	1406	Laetitia Fartoux	CPP Sud Ouest et Outre Mer III<br>Hopital Pellegrin-service pharmacologie Clinique Bat 1A<br>Place Amelie Raba Leon<br>Bordeaux 33076 Cedex
France (FRA)	1407	Julien Forestier	CPP Sud Ouest et Outre Mer III<br>Hopital Pellegrin-service pharmacologie Clinique Bat 1A<br>Place Amelie Raba Leon<br>Bordeaux 33076 Cedex
France (FRA)	1408	Jean Didier Grange	CPP Sud Ouest et Outre Mer III<br>Service Pharmacologie Clinque Bat 1A Hopital Pellegrin<br>Place Amelie Raba Leon<br>Bordeaux 33076 Cedex
France (FRA)	1409	Philippe Mathurin	CPP Sud Ouest et Outre Mer III<br>Service Pharmacologie Clinque Bat 1A Hopital Pellegrin<br>Place Amelie Raba Leon<br>Bordeaux 33076 Cedex
France (FRA)	1410	Eric Nguyen-Khac	CPP Sud Ouest et Outre Mer III<br>Hopital Pellegrin-service pharmacologie Clinique Bat 1A<br>Place Amelie Raba Leon<br>Bordeaux 33076 Cedex

COUNTRY	SITE	PRINCIPAL INVESTIGATOR	IRB / IEC INFORMATION
France (FRA)	1412	Albert Tran	CPP Sud Ouest et Outre Mer III<br>Hopital Pellegrin-service pharmacologie Clinique Bat 1A<br>Place Amelie Raba Leon<br>Bordeaux 33076 Cedex
France (FRA)	1413	Julien Vergniol	CPP Sud Ouest et Outre Mer III<br>Service Pharmacologie Clinque Bat 1A Hopital Pellegrin<br>Place Amelie Raba Leon<br>Bordeaux 33076 Cedex
France (FRA)	1414	Jean-Marie Peron	CPP Sud Ouest et Outre Mer III<br>Service Pharmacologie Clinque Bat 1A Hopital Pellegrin<br>Place Amelie Raba Leon<br>Bordeaux 33076 Cedex
Italy (ITA)	1501	Fabio Piscaglia	Comitato Etico Indipendente dell’Azienda Ospedaliero-Universitaria Policlinico S. Orsola Malpighi<br>Via Albertoni, 15<br>Bologna 40138
Italy (ITA)	1502	Stefano Cascinu	Comitato etico dell’azienda ospedaliero universitaria Ospedali Riuniti Umberto I G.M. Lancisi<br>Via Conca, 71<br>Torrette di Ancona, Ancona 60126
Italy (ITA)	1503	Giovan Giuseppe Di Costanzo	Comitato Etico dell’Azienda Ospedaliera Antonio Cardurelli di Napoli<br>Via Cardarelli, 9<br>Napoli 80131
Italy (ITA)	1504	Antonio Gasbarrini	Comitato Etico dell’Universita Cattolica del Sacro Cuore – Policlinico Universitario Agostino Gemelli<br>Largo Agostino Gemelli, 8<br>Roma 00168
Italy (ITA)	1505	Antonio Avallone	Comitato etico dell’IRCCS Istituto Nazionale per lo Studio e la Cura dei Tumori Fondazione G. Pascale<br>Via Mariano Semmola, I<br>Napoli 80131
Italy (ITA)	1507	Nicola Silvestris	Ethic Committee IRCCS Cancer Institute Giovanni Paolo II<br>Via Orazio Flacco 65<br>Bari 70124

COUNTRY	SITE	PRINCIPAL INVESTIGATOR	IRB / IEC INFORMATION
Italy (ITA)	1508	Antonio Craxi	Comitato Etico dell'Azienda Ospedaliera Universitaria Paolo Giaccone<br>Via del Vespro 127<br>Palermo 90127
Italy (ITA)	1509	Bruno Daniele	Comitato Etico dell'Azienda Ospedaliera G. Rummo<br>Oncologia Medica<br>Via dell'Angelo, 1<br>Benevento 82100
Poland (POL)	1601	Jacek Jassem	Niezalezna Komisja Bioetyczna do Spraw Badan Naukowych przy Gdanskim Uniwersytecie Medycznym<br>ul. M. Sklodowskiej-Curie 3a<br>Gdansk 80-210
Poland (POL)	1602	Krzysztof Simon	Niezalezna Komisja Bioetyczna do Spraw Badan Klinicznych przy Gdanskim Uniwersytecie Medycznym<br>ul. Marii Sklodowskiej-Curie 3a<br>Gdansk 80-210
Poland (POL)	1603	Lucjan Wyrwicz	Nizalezna komisja Bioetyczna do Spraw Badan Naukowych przy Gdanskim Uniwersytecie Medycznym<br>ul. Marii Sklodowskij-Curii 3a<br>Gdansk 80 210
United Kingdom (GBR)	1701	Debashis Sarker	NRES Committee London – Westminster Research Health Authority, Ground Floor, Skipton House<br>80 London Road<br>London SE1 6LH
United Kingdom (GBR)	1702	Thomas Ronald Jeffry Evans	NRES Committee London – Westminster Research Health Authority, Ground Floor, Skipton House<br>80 London Road<br>London SE1 6LH
United Kingdom (GBR)	1703	Daniel Harrison Palmer	NRES Committee London – Westminster Research Health Authority, Ground Floor, Skipton House<br>80 London Road<br>London SE1 6LH
United Kingdom (GBR)	1704	Yuk Ting Ma	NRES Committee London – Westminister Research Health Authority, Ground Floor, Skipton House<br>80 London Road<br>London SE1 6LH

COUNTRY	SITE	PRINCIPAL INVESTIGATOR	IRB / IEC INFORMATION
United Kingdom (GBR)	1705	Harpreet Singh Wasan	NRES Committee London – Westminster<br>Research Health Authority, Ground Floor, Skipton House<br>80 London Road<br>London SE1 6LH
United Kingdom (GBR)	1706	Tim Meyer	NRES Committee London – Westminster<br>Level 3, Block B<br>Whitefriars, Lewins Mead<br>Bristol BS1 2NT
United Kingdom (GBR)	1707	Richard Anthony Hubner	NRES Committee London – Westminister<br>Level 3, Black B<br>Whitefriars, Lewins Mead<br>Bristol BS1 2NT
Germany (DEU)	1802	Dirk Waldschmidt	Ethikkommission der Medizinischen Hochschule Hannover<br>Carl-Neuberg-Strasse 1<br>Hannover, Niedersachsen 30625
Germany (DEU)	1804	Arndt Weinmann	Ethikkommission der Medizinischen Hochschule Hannover<br>Carl-Neuberg-Strasse 1<br>Hannover, Niedersachsen 30625
Germany (DEU)	1805	Arndt Vogel	Ethikkommission der Medizinischen Hochschule Hannover<br>Carl-Neuberg-Strasse 1<br>Hannover, Niedersachsen 30625
Germany (DEU)	1807	Christoph Springfeld	Ethikkommission der Medizinischen Hochschule Hannover<br>Carl-Neuberg-Strasse 1<br>Hannover, Niedersachsen, 30625
Germany (DEU)	1808	Stefan Pluntke	Ethikkommission der Medizinischen Hochschule Hannover<br>Carl-Neuberg-Strasse 1<br>Hannover, Niedersachsen 30625
Germany (DEU)	1809	Michael Bitzer	Ethikkommission der Medizinischen Hochschule Hannover<br>Carl-Neuberg-Strasse 1<br>Hannover, Niedersachsen 30625
Russian Federation (RUS)	1901	Valery V. Breder	Ethical Council at the Ministry of Health of Russian Federation<br>3, Rakhmanovsky lane<br>Moscow 127994

COUNTRY	SITE	PRINCIPAL INVESTIGATOR	IRB / IEC INFORMATION
Russian Federation (RUS)	1902	Dmitriy Komov	Ethical Council at the Ministry of Health of Russian Federation<br>3, Rakhmanovsky lane<br>Moscow 127994
Russian Federation (RUS)	1903	Oleg N. Lipatov	Ethical Council at the Ministry of Health of Russian Federation<br>3, Rakhmanovsky lane<br>Moscow 127994
Russian Federation (RUS)	1904	Maria M. Konstantinova	Ethical Council at the Ministry of Health of Russian Federation<br>3, Rakhmanovsky lane<br>Moscow 127994
Russian Federation (RUS)	1905	Marina N. Nachaeva	Ethical Council at the Ministry of Health of Russian Federation<br>3, Rakhmanovsky lane<br>Moscow 127994
Russian Federation (RUS)	1906	Anna A. Kochatkova	Ethical Council at the Ministry of Health of Russian Federation<br>3, Rakhmanovsky lane<br>Moscow 127994
Russian Federation (RUS)	1907	Georgy M. Manikhas	Ethical Council at the Ministry of Health of Russian Federation<br>3, Rakhmanovsky lane<br>Moscow 127994
Belgium (BEL)	2001	Jean Delwaide	Ethisch Comite Universitair Ziekenhuis Antwerpen<br>Wilrijkstraat 10<br>Edegem 2650
Belgium (BEL)	2002	Sven Francque	Universitair Ziekenhuis Antwerpen<br>Wilrijkstraat 10<br>Edegem 2650
Belgium (BEL)	2003	Jean-Luc Van Laethem	Ethisch Comite Universitair Ziekenhuis Antwerpen<br>Wilrijkstraat 10<br>Edegem 2650
Spain (ESP)	2101	Mariona Calvo Campos	Hospital Universitario Marques de Valdecilla<br>Avda. de Valdecilla s/n<br>Santander 39008
Spain (ESP)	2102	Juan Ignacio Delgado	Hospital Universitario Marques de Valdecilla<br>Avda. de Valdecilla s/n<br>Santander Cantabria, 39008
Spain (ESP)	2103	Carlos Lopez Lopez	Hospital Universitario Marques de Valdecilla<br>Avda. de Valdecilla s/n<br>Santander 39008

COUNTRY	SITE	PRINCIPAL INVESTIGATOR	IRB / IEC INFORMATION
Spain (ESP)	2104	Carlos Gomez Martin	Hospital Universitario Marques de Valdecilla<br>Avda. de Valdecilla s/n<br>Santander 39008
Spain (ESP)	2105	Javier Sastre	Hospital Universitario Marques de Valdecilla<br>Avd. de Valdecilla s/n<br>Santander 39008
Spain (ESP)	2106	Andres Munoz Martin	Hospital Universitario Marques de Valdecilla<br>Avda. de Valdecilla s/n<br>Santander 39008
Spain (ESP)	2107	Raquel Guardeno	Comite Etico de Investigacion Clinica Cantabria<br>Avda. Cardenal Herrera Oria s/n<br>Edificio IFIMAV, 3[a] Planta<br>Madrid 39011
Spain (ESP)	2108	Agustin Albillos Martinez	Comite Etico de Investigacion Clinica Cantabria<br>Avda. Cardenal Herrera Oria s/n<br>Edificio IFIMAV, 3[a] Planta<br>Madrid 39011
Canada (CAN)	2202	Rakesh Goel	The Ottawa Health Science Network Research Ethics Board<br>725 Parkdale Ave<br>Ottawa, ON K1Y 4E9
Canada (CAN)	2204	Petr Kavan	McGill Faculty of Medicine – Institutional Review Board<br>3655 Promenade Sir William Osler, # 633<br>Montreal, Quebec H3G 1Y6
China (CHN)	2401	Shukui Qin	Ethic Committee of Nanjing Bayi Hospital<br>No. 34 Yang Gong Jing 34 Biao, Baixia District<br>Nanjing, Jiangsu 210002
China (CHN)	2402	Ying Cheng	Ethics Committee of Jilin Province Cancer Hospital<br>No. 1018, Huguang Rd, Chaoyang District<br>Changchun, Jilin 130012
China (CHN)	2403	Yuxian Bai	Ethic Committee of hrbin Medical University Cancer Hospital<br>No. 50 Haping Road, Nangang District<br>Harbin, Heilongjia 150081
China (CHN)	2404	Helong Zhang	IEC of Tangdu Hospital, Fourth Military Medical University<br>No. 1, Xinsi Road, Baqiao District<br>Xi’an, Shaanxi 710038
China (CHN)	2405	Hailan Lin	IRB of Fujian Province Cancer Hospital<br>No. 420, Fuma Road, Fuzhou, Fujian Province<br>Fuzhou, Fujian 350014

COUNTRY	SITE	PRINCIPAL INVESTIGATOR	IRB / IEC INFORMATION
China (CHN)	2407	Min Tao	Ethical Committee for drug clinical trial of The First Affiliated Hospital of Soochow University<br>No. 188 Shizi Street<br>Suzhou, Jiangsu 215006
China (CHN)	2408	Houjie Liang	EC of First Affiliated Hospital, Third Military Medical University<br>No. 30 Gaotanyanzheng Street, Shapingba District, Chongqing 400038
China (CHN)	2409	Jianming Xu	EC of 307 Hospital of PLA<br>No. 8, East Street, Fengtai District<br>Beijing 100071
China (CHN)	2410	Tianqiang Song	IEC of Tianjin Medical University Cancer Institute and Hospital<br>Huanhuxi Road, Hexi District<br>Tianjin 300060
China (CHN)	2411	Guohong Han	Ethics Committee of Xijing Hospital<br>No. 127, Changle West Road, Xi’an City, Shaanxi Province<br>Xi’an, Shaanxi 710032
China (CHN)	2412	Zhiqiang Meng	Ethic Committee of Fudan University Shanghai Cancer Center<br>No. 270 Dong’an Road, Xuhui District<br>Shanghai 200032
China (CHN)	2413	Xuenong Ouyang	Ethic Committee, Nanjing Military Fuzhou General Hospital<br>No. 156 Xi’er Huan North Road, Fujian Province<br>Fuzhou, Fujian 350025
China (CHN)	2414	Jiejun Wang	Ethic Committee of Shanghai Changzheng Hospital<br>No 415 Fengyang Road, Huangpu District<br>Shanghai 200003
China (CHN)	2415	Zhenggang Ren	Ethic Committee, Zhongshan Hospital, Fudan University<br>No. 180, Fenglin Road<br>Shanghai 200032
China (CHN)	2416	Zhendong Chen	Ethics Committee of The Second Affiliated Hospital of Anhui Medical University<br>No. 678 Furong Road, Economic and Technological Development Zone<br>Hefei, Anhui 230601
China (CHN)	2417	Bangwei Cao	The Ethical Committee of Beijing Friendship Hopsital, Capital Medical University<br>No. 36, Yong’an Road<br>Beijing 100050

COUNTRY	SITE	PRINCIPAL INVESTIGATOR	IRB / IEC INFORMATION
China (CHN)	2418	Pan Hongming	Ethic Committee, Sir Runrun Shaw Hospital, School of Medicine, Zhejiang University<br>No. 3 East Qing Chun Road<br>Hangzhou, Zhejiang 310016
China (CHN)	2419	Lin Shen	Ethical Committee of Beijing Cancer Hospital<br>No. 52 Fucheng Road, Haidian District<br>Beijing 100142
China (CHN)	2420	Baocheng Wang	Ethic Committee of Jinan Military General Hospital<br>No. 25, Shifan Road, Tianqiao District<br>Jinan, Shandong 250031
China (CHN)	2421	Jiwei Liu	Ethic Committee of First Affiliated Hospital of Dalian Medical University<br>No. 222 Zhongshan Road, Xigang District<br>Dalian, Liaoning 116011
China (CHN)	2422	Minshan Chen	Ethical Committee of Sun Yat-sen University Cancer Center<br>651 Dongfeng East Road<br>Guangzhou, Guangdong China 510060
China (CHN)	2423	Shanzhi Gu	Ethic Committee of Hunan Cancer Hospital<br>No. 283 Tongzipo Road, Yuelu District<br>Changsha, Hunan 410006
S. Korea (KOR)	3001	Jung-Hwan Yoon	Seoul National University Hospital IRB<br>101 Daehak-ro, Jongno-gu<br>Seoul 03080
S. Korea (KOR)	3002	Jong-Young Choi	The Catholic University of Korea Seoul St. Mary's Hospital Institutional Review Board<br>222, Banpo-Daero, Seocho-gu<br>Seoul 06591
S. Korea (KOR)	3003	Kwan Sik Lee	Gangnam Severance Hospital, Yonsei University Health System Institutional Review Board<br>211 Eonju-ro, Gangnam-gu<br>Seoul 06273
S. Korea (KOR)	3004	Jee Hyun Kim	Seoul National University Bundang Hospital IRB<br>82, Gumi-ro 173beon-gil, Bundang-gu<br>Seongnam-si, Gyeonggi-do 463-707
S. Korea (KOR)	3005	Kwang-Hyub Han	Severance Hospital Institutional Review Board, Yonsei University Health System<br>50-1 Yonsei-ro, Seodaemun-gu<br>Seoul 03722
S. Korea (KOR)	3006	Jeong Heo	Pusan National University Hospital Insitutional Review Board<br>179 Gudeok-Ro, Seo-Gu<br>Busan 49241

COUNTRY	SITE	PRINCIPAL INVESTIGATOR	IRB / IEC INFORMATION
S. Korea (KOR)	3007	Joong-Won Park	National Cancer Center Institutional Review Board<br>323 Ilsan-ro, Ilsandong-gu<br>Goyang-si, Gyeonggi-do 10408
S. Korea (KOR)	3008	Seung Woon Paik	Samsung Medical Center Institutional Review Board<br>81, Irwon-ro, Gangnam-gu<br>Seoul 06351
S. Korea (KOR)	3009	Baek-Yeol Ryoo	Asan Medical Center IRB<br>88, Olaypic-ro 43-gil, Songpa-gu<br>Seoul 05505
S. Korea (KOR)	3010	Byung-Ho Kim	Kyung Hee University Hospital Institutional Review Board<br>23, Kyungheedae-ro, Dongdaemun-gu<br>Seoul 130-872
S. Korea (KOR)	3011	Youn-Jae Lee	Inje University Busan Paik Hospital IRB<br>75 Bokji-ro, Busanjin-gu<br>Busan 47392
S. Korea (KOR)	3012	Won Young Tak	Institutional Review Board of Kyungpook National University Hospital<br>135 Dongduk-ro, Jung-gu<br>Daegu 41944
S. Korea (KOR)	3013	Soon Ho Um	Korea University Anam Hospital Institutional Review Board<br>73, Inchon-ro, Seongbuk-gu<br>Seoul 02841
S. Korea (KOR)	3014	Young-Eun Joo	Chonnam National University Hwasun Hospital Institutional Review Board<br>322 SeoYang-Ro, Hwasun-Eup<br>Hwasun-Gun, Jeonnam 58128
S. Korea (KOR)	3015	Jung Hyun Kwon	The Catholic University of Korea Incheon St. Mary’s Hospital Institutional Review Board<br>56 Dongsu-ro, Bupyeong-gu<br>Incheon 403-720
S. Korea (KOR)	3016	Byoung Yong Shim	The Catholic University of Korea St. Vincent’s Hospital IRB<br>93 Jungbu-daero, Paldal-gu<br>Suwon 16247
S. Korea (KOR)	3017	SangYoung Han	Dong-A University Hospital Institutional Review Board<br>26 Daeshingongwon-ro, Seo-gu<br>Busan 49201

COUNTRY	SITE	PRINCIPAL INVESTIGATOR	IRB / IEC INFORMATION
S. Korea (KOR)	3018	Seong Gyu Hwang	CHA Bundang Medical Center, CHA University Institutional Review Board<br>59 Yatap-ro, Bundang-gu<br>Seongnam-si, Gyeonggi-do 463-712
Thailand (THA)	3201	Tawesak Tanwandee	Siriraj Institution Review board (SIRB), Faculty of Medicine, Siriraj Hospital, Mahidol University<br>His Majesty the King's 80th Birthday Anniversary 5th December<br>2nd Floor, Room no. 210, 2 Wang Lang Road<br>Bangkok-noi, Bangkok 10700
Thailand (THA)	3202	Abhasnee Sobhonslidsuk	Ethical Clearance on Human Rights Related to Researcher Involving Human Subjects<br>Faculty of Medicine, Ramathibodi Hospital.<br>Mahidol University, 270 Rama VI Road<br>Ratchathewee, Bangkok 10400
Thailand (THA)	3203	Ekkapong Tharavichitkul	Institute for the Development of Human Research Protections, Department of Medical Science Ministry of Public Health<br>Room 702-703, Building 8, Floor 7<br>Muang, Nonthaburi 11000
Thailand (THA)	3204	Chaiyut Charoentum	Research Ethics Committee, Faculty of Medicine, Chiang Mai University<br>110 Intavaroros Road, Sriphoom<br>Muang, Chiang Mai 50200
Thailand (THA)	3205	Piyawat Komolmit	The Institutional Review Board, Faculty of Medicine Chulalongkorn University<br>King Chulalongkorn Memorial Hospital<br>3rd Floor, Anantamahidol Building<br>1873 Rama IV Road<br>Patumwan, Bangkok 10330
Israel (ISR)	4301	Salomon M. Stemmer	Ethics Committee, Rabin Medical Center<br>39 Jabotinski St.<br>Petah Tikva 49100
Singapore (SGP)	4401	Akhil Chopra	National Healthcare Group, Domain Specific Review Board (NHG DSRB)<br>c/o National Healthcare Group. Research & Development Office<br>Nexus @One-North (South Tower), No. 3 Fusionopolis Link<br>Singapore 138543
Singapore (SGP)	4403	Chee Cheng Ean	National Healthcare Group, Domain Specific Review Board (NHG DSRB)<br>6 Commonwealth Lane , Level 6 GMTI Building<br>Singapore 149547

COUNTRY	SITE	PRINCIPAL INVESTIGATOR	IRB / IEC INFORMATION
Singapore (SGP)	4404	Wen Son Hsieh	Parkway Independent Ethics Committee<br>c/o Parkway Pantai Limited<br>TripleOne Somerset 111, Somerset Road #15-01<br>Singapore 238164
Singapore (SGP)	4405	Choo Su Pin	SingHealth Centralised Institutional Review Board (CIRB)<br>Singapore Health Services Pte Ltd<br>31 Third Hospital Avenue<br>#03-03 Bowyer Block C<br>Singapore 168753
Taiwan (TWN)	4501	Chia-Jui Yen	Institutional Review Board, National Cheng Kung University Hospital<br>138, Sheng-Li Rd.<br>Tainan, Taiwan (R.O.C.) 704
Taiwan (TWN)	4502	Cheng-Yao Lin	Institutional Review Board of Chi-Mei Medical Center<br>No. 901, Zhonghua Rd., Yongkang Dist.<br>Tainan, Taiwan (R.O.C.) 710
Taiwan (TWN)	4503	Teng-Yu Lee	Institutional Review Board, Taichung Veterans General Hospital<br>1650 Taiwan Boulevard Sect. 4<br>Taichung, Taiwan 40705
Taiwan (TWN)	4504	Tsai-Sheng Yang	Chang Gung Medical Foundation Institutional Review Board<br>199, Tung Hwa North Road<br>Taipei, Taiwan (R.O.C.) 10507
Taiwan (TWN)	4505	Yee Chao	Institutional Review Board, Taipei Veterans General Hospital<br>No. 201, Sec. 2, Shih-Pai Road<br>Taipei, Taiwan R.O.C. 11217
Taiwan (TWN)	4506	Ann-Lii Cheng	Research Ethics Committee of National Taiwan University Hospital<br>7 Chung-Shan South Road<br>Taipei, Taiwan R.O.C. 10002
Taiwan (TWN)	4507	Kun-Ming Rau	Chang Gung Medical Foundation Institutional Review Board<br>199, Tung Hwa North Road<br>Taipei, Taiwan (R.O.C.) 10507
Taiwan (TWN)	4508	Sien-Sing Yang	Institutional Review Board of the Cathay General Hospital<br>No. 280, Jen-Ai Rd., Sec. 4<br>Taipei, Taiwan (R.O.C.) 10630

COUNTRY	SITE	PRINCIPAL INVESTIGATOR	IRB / IEC INFORMATION
Taiwan (TWN)	4509	Tsang-En Wang	Mackay Memorial Hospital Institutional Review Board<br>92 Sec. 2 Chungshan N. Rd.<br>Taipei, Taiwan (R.O.C.) 10049
Taiwan (TWN)	4510	Shinn-Cherng Chen	Institutional Review Board, Kaohsiung Medical University Chung-Ho Memorial Hospital<br>No. 100, Tzyou 1st Road<br>Kaohsiung, Taiwan (R.O.C.) 807
Taiwan (TWN)	4511	Long-Bin Jeng	Research Ethics Committee, China Medical University Hospital<br>2 Yude Road<br>Taichung, Taiwan (R.O.C.) 40447
Taiwan (TWN)	4512	Yu-Lueng Shih	Institution Review Board, Tri-Service General Hospital<br>No. 325, Sec. 2, Cheng-Kung Road, Neihu<br>Taipei, Taiwan R.O.C. 11490
Philippines (PHL)	4601	Marilyn O. Arguillas	Davao Doctors Hospital Institutional Ethics Review Board<br>118 E. Quirino Avenue<br>Davao 8000
Philippines (PHL)	4602	Arlene M. Kuan	Vicente Sotto Memorial Medical Center Ethics Review Committee<br>B. Rodriguez St.<br>Cebu 6000
Philippines (PHL)	4603	Judy Y. Lao-Tan	Cebu Doctors University Hospital Research Ethics Committee<br>G/F Administrative Building, Gov. M. Roa St., Osmena Boulevard<br>Cebu 6000
Philippines (PHL)	4604	Maximo De Guzman Bello III	St. Luke’s Medical Center Ethics Review Committee<br>Annex 3, 5th Floor<br>279 E. Rodriguez Sr. Avenue<br>Quezon City, NCR 1102
Philippines (PHL)	4605	Annielyn Beryl Ong-Cornel	Veterans Memorial Medical Center Institutional Review Board<br>North Avenue, Diliman<br>Quezon City, NCR, 0870
Hong Kong (HKG)	4701	Stephen Lam Chan	Joint Chinese University of Hong Kong – New Territories East Cluster Clinical Research Ethics Committee<br>8th Floor, Lui Che Woo Clinical Science Building<br>Prince of Wales Hospital, 30-32 Ngan Shing Street, Shatin, N.T.

COUNTRY	SITE	PRINCIPAL INVESTIGATOR	IRB / IEC INFORMATION
Hong Kong (HKG)	4702	Ann-Shing Lee	NTW Cluster Clinical & Research Ethics Committee<br>Room 5.130, 5/F, Rehabilitation Block<br>Tuen Mun Hospital<br>Tuen Mun
Hong Kong (HKG)	4703	Thomas Chung Cheung Yau	Institutional Review Board of the University of Hong Kong/Hospital Authority Hong Kong West Cluster<br>Room 901, Administration Block, Queen Mary Hospital, 102 Pokfulam Road<br>Pokfulam
Hong Kong (HKG)	4704	Kai Cheong Roger Ngan	Research Ethics Committee (Kowloon Central/Kowloon East)<br>Room 712, Block S, Queen Elizabeth Hospital<br>30 Gascoigne Road<br>Kowloon
Malaysia (MYS)	4801	Khean Lee Goh	Medical Ethics Committee<br>University Malaya Medical Centre<br>Lembah Pantai, Kuala Lumpur 59100
Malaysia (MYS)	4802	Aloysius Raj Thangadorai	Joint Penang Independent Ethics Committee<br>The JPEC Secretariat c/o Info Kinetics Clinical Research Centre<br>3rd Floor, Gleneagles Medical Centre<br>1, Jalan Pangkor<br>Penang 10050
Malaysia (MYS)	4803	Chee Ming Wong	Medical Research Ethics Committee (MREC), Ministry of Health c/o National Institutue of Health Secretariat<br>Institute of Health Management, Jalan Rumah Sakit<br>Bangsar, Kuala Lumpur 59000
Malaysia (MYS)	4804	Tee Hoi Poh	Medical Research Ethics Committee (MREC), Ministry of Health c/o National Institute of Health Secretariat<br>Institute Health Management, Jalan Rumah Sakit<br>Bangsar, Kuala Lumpur 59100
Malaysia (MYS)	4805	Kin Foong Lim	Medical Research Ethics Committee (MREC), Ministry of Health c/o National Institute of Health Secretariat<br>Institute Health Management, Jalan Rumah Sakit<br>Bangsar, Kuala Lumpur 59000

16.1.3.2 Sample Informed Consent Form

The most current version of the master global sample informed consent form issued during this study is provided on the following pages. Previous versions are available upon request.

- Master ICF Template version 8.1 dated 15 May 2016

INFORMED CONSENT FORM

AGREEMENT TO BE IN A RESEARCH STUDY

STUDY SPONSORS/ LOCATION:	Eisai Inc. ("Sponsor")<br>100 Tice Boulevard<br>Woodcliff Lake, New Jersey 07677<br><br>Eisai Ltd. ("Sponsor")<br>European Knowledge Centre<br>Mosquito Way<br>Hatfield, Hertfordshire<br>AL10 9SN UK<br><br>Eisai Co., Ltd. ("Sponsor")<br>4-6-10 Koishikawa<br>Bunkyo-Ku,<br>Tokyo 112 8088<br>Japan
STUDY NUMBER:	E7080-G000-304
NAME OF STUDY:	A Multicenter, Randomized, Open-Label, Phase 3 Trial to Compare the Efficacy and Safety of Lenvatinib (E7080) Versus Sorafenib in First-Line Treatment of Subjects With Unresectable Hepatocellular Carcinoma
STUDY DOCTOR:	TBD
IRB/ EC NUMBER:	TBD
IRB APPROVAL DATE:	TBD
Subject's Name (Printed):	________________________ *[initials may be used]*

Introduction:

You have been invited to take part in a clinical research study. Before you decide whether to take part, it is important for you to understand why this research is being done, what it will involve and how it will affect your daily life. Please take time to read the following information carefully and discuss it with your family, friends or anyone else with whom you may want. If you need more information, please ask your study doctor. Please take your time in deciding if you want to participate in this study.

If you agree to participate, the study doctor may ask your permission to let your family and/or regular doctor know of your decision.

Why is this Study Being Done?

This study is being done by a company that does drug research studies (called a "Sponsor"). The name of the company is Eisai.

Lenvatinib (E7080) is a drug that is being developed for the treatment of cancer, including unresectable hepatocellular carcinoma, which is a common type of liver cancer.

The main purpose of this study is to test the overall survival effects of lenvatinib patients with your type of liver cancer. In order to evaluate lenvatinib, this study will be comparing lenvatinib against sorafenib (Nexavar®).

Sorafenib is a Food and Drug Administration (FDA) approved and European Commission approved drug for the treatment of your type of advanced liver cancer (hepatocellular carcinoma), as well as for kidney cancer (advanced renal cell carcinoma).

The purpose of the study is to see if there is any shrinkage of your tumor and for how long any effect on your tumor lasts. The study will also collect information on how long you live and the side effects that you may experience during the study.

Blood tests will be drawn only from subjects taking lenvatinib to look at the levels of lenvatinib in blood. These samples are called "pharmacokinetic samples." The sponsor, the doctor and staff will know what study drug you are receiving and whether you will be having these blood tests drawn.

What is the Status of the Drug[s] Involved in this Study?

Lenvatinib is a drug designed to work by stopping the formation of new blood vessels that help tumors to grow and spread. Levantinib is an "Investigational" Study Drug. "Investigational" means that the drug being tested in this study has not been approved by the FDA, or any Health Authority throughout the world for the treatment of any type of cancer. However, the FDA has authorized the conduct of this study for purposes of learning more about the safety and effectiveness of the investigational drug. Study drug will be provided free of charge during the study.

It is not known whether the study drug will have an effect on your type of liver cancer compared to what is normally expected for liver cancer with other treatments.

Information describing this study will also be included in a clinical trial registry data bank. The public will be able to read this information using the internet.

As of 12 Feb 2016 there have been approximately 34 other studies testing lenvatinib in about 2,000 research subjects with different types of cancers including your type of liver cancer (hepatocellular carcinoma) in Europe, the USA and Japan. One of the studies testing lenvatinib was a smaller study that enrolled 46 research subjects who were all diagnosed with hepatocellular carcinoma. Fourteen study sites (12 sites in Japan and 2 sites in Korea) participated in the study.

Based on investigator's assessment, lenvatinib was shown to demonstrate activity in subjects with hepatocellular carcinoma, which warrants testing lenvatinib in this larger trial.

Why am I Being Asked to Join this Study?

You are being asked to take part in this study because you have been diagnosed as having unresectable hepatocellular carcinoma, that cannot be removed by surgery and has not been previously treated.

Where Else is this Study Being Done and How Long Will the Study Take?

This study will be done at about 150 research centers located in Asia Pacific Region, Europe and North America. The study will include approximately 940 subjects with unresectable hepatocellular carcinoma.

We expect that the study will take about 36 months to complete.

The results from this study should be known approximately 24 months after the last subject is enrolled.

How Long Will I Be in the Study?

If you agree to join this study, and you meet all of the study entry requirements, you will continue on study unless:

1) your disease gets worse;
2) you have unacceptable side effects;
3) you decide that you no longer wish to take the study drug;
4) the study is stopped by the Sponsor, or Health Authority for any reason;
5) you fail to follow instructions given by the study doctor; or
6) the study doctor believes it is best for you to no longer be in the study.

What if I Decide Not to Take Part in this Study?

Taking part in this research study is voluntary. You may choose not to take part. If you do choose to be in the study, you may change your mind at any time. If you do not want to enter the study or decide to stop being in the study, your relationship with the study staff will not change, and there will be no penalty to you. You will not lose any benefits including health care services to which you are otherwise entitled.

What Will Happen If I Agree to Take Part in this Study?

To find out if you can take part in this study and after you have signed this consent form, you will go through a screening process. In this process, you will be asked about your general health and your medical/surgical history. You will also be asked about medicines, prescriptions and any over the counter drugs and supplements you are taking right now or have taken during the past 30 days before the Screening Visit. Regardless of whether you are assigned to lenvatinib or sorafenib, you will undergo all the same screening procedures and tests.

Screening Tests and Procedures

Once all your questions have been answered and you feel comfortable that you understand what this study involves you will need to sign this informed consent. You will have the following tests and procedures to determine if you are eligible to take part in this study. If any of these tests were performed prior to signing consent, as part of your routine care, and if they fall within the time allowed by the study protocol, they may be used and need not to be repeated. This evaluation process may take up to a maximum of three weeks and will include the following:

- Assessment of your ability to do daily activities
- Assessment to link the stage of your disease. This will be a factor to determine which treatment you will be assigned to
- Questions about your medical & surgical history
- Viral test to see if you have infection-causing viruses of hepatitis or HIV (HIV Ab, HCV Ab, HBsAg) (4mL or less than 1 teaspoon of blood will be taken)
- Examination to see the inside lining of your digestive tract (gastroenterological endoscopy)
- Complete physical exam including height, weight and vital signs (blood pressure, heart rate, temperature, and breathing rate)
- Heart Tests:
  - Electrocardiogram (ECG) - An ECG records the rhythms and electrical activity of your heart. A number of electrodes (small, sticky patches) are stuck on your arms, legs and chest. The electrodes are connected to a machine that records the electrical signals of each heartbeat and monitors how your heart is working.
  - Echocardiogram or MUGA Scan - An echocardiogram is an ultrasound scan of the heart. Ultrasound is a very high frequency sound that you cannot hear, but it can be emitted and detected by special machines and is used to build up a detailed picture of the heart. You will be lying down on a bed and a small doppler (with gel on it) will reflect pictures onto a screen of how your heart is pumping. This test gives good information about the structure and function of your heart. A MUGA Scan (Multi Gated Acquisition Scan) is a nuclear medicine test used to evaluate the function of the heart ventricles (pumping chambers). The MUGA test involves the injection of a radioactive marker into the bloodstream. A scanner is then used to provide a movie-like image of the beating heart, which allows the doctor to determine the health of the heart's major pumping
- Blood tests (total of about 1-2 teaspoons or 5-10 mL of blood will be taken):
  - Chemistry (test that look at your kidney, liver, thyroid function and blood salts),
  - Hematology (test looking at the amount of red blood cells – cells that carry oxygen, white blood cells – cells that fight infection, and platelets – cells that help you form clots, in your blood)
  - Urine or blood pregnancy test (if you are a woman who is able to become pregnant) Women must have a negative pregnancy test in order to take part in this study
- Urine test to check for any protein or sugar
- Test of liver cirrhosis, result of liver disease that could lead to loss of liver function. (Child-Pugh score test)
- Blood clotting exam to test for bleeding problems (2.7 mL or less than 1 teaspoon of blood will be taken)
- Special chemistry test to check for liver and thyroid gland problems (8.7 mL or about 2 teaspoons of blood will be taken)

- Assessment of your disease to find out the extent of your cancer:
  - CT scan(s) and/or MRI(s), of brain, chest, abdomen and pelvis. Also, CT scan(s) or routine MRI(s), and/or x-ray(s) of your cancer site(s). CT stands for computerized tomography. A CT scanner is used to take a series of X-rays of your body at slightly different angles. A computer puts these together to produce a very detailed picture of the inside of your body. Magnetic resonance imaging (MRI) uses a strong magnetic field and radio waves to produce detailed pictures of the inside of your body. The pictures produced by the CT scans and the MRIs provide doctors with information to help them assess the extent of your cancer

- You will also be asked about other medications you are taking and if you have any side effects from those medications

If it is determined that you are eligible to take part in the study you will be asked to return to the clinic the day before your scheduled start date (first day of receiving treatment) - this visit is called the Baseline Visit. If all the tests listed above were not completed during the screening period, or if they were done beyond the time allowed by the study, you will be asked to have some of the tests listed above repeated including blood draws.

At the Baseline Visit you may have the following tests done:

- Assessment of your ability to do daily activities
- Questions about your medical & surgical history
- Vital signs: blood pressure, heart rate, temperature, breathing rate, including weight
- Complete physical examination if the screening examination was performed more than 7 days before your Cycle 1 Day 1 visit date
- Blood tests (total of about 1-2 teaspoons or 5 -10 mL of blood will be taken):
  - Chemistry (test that look at your kidney, liver, thyroid function and blood salts),
  - Hematology (test looking at the amount of red blood cells – cells that carry oxygen, white blood cells – cells that fight infection, and platelets – cells that help you form clots, in your blood)
  - Urine or blood pregnancy test (if you are a woman who is able to become pregnant). Women must have a negative pregnancy test
- Urine test to check for any protein or sugar
- Test of liver cirrhosis, result of liver disease that could lead to loss of liver function. (Child-Pugh score test)
- Blood clotting exam to test for bleeding problems (2.7 mL or less than 1 teaspoon of blood will be taken)
- Questionnaires to test your general health and quality of life called EORTC-QLQ-C30, EORTC-QLQ-HCC18 and EQ-5D
- You will also be asked about other medications you are taking and if you have any side effects from those medications

Cycle 1, Day 1:

Assignment into Your Study Group

If you agree to be in this clinical study, and after screening, you meet all of the study entry requirements, you will be assigned to receive either lenvatinib or sorafenib. The following four factors will determine which drug you will receive:

1) Your geographic location

2) The extent of your disease including if the disease has spread beyond the liver or extends into critical blood vessels (macroscopic portal vein invasion)

3) Your ability to do daily activities

4) Your body weight

You and your doctor and staff will know if you will receive lenvatinib or sorafenib.

What will happen once I am assigned to receive either lenvatinib or sorafenib?

Depending on your weight, orally (by mouth) you will take 8 mg or 12 mg of lenvatinib once a day every day or 400 mg of sorafenib twice daily for as long as you are on study and receiving benefit.

- Lenvatinib comes in 4 mg capsule strengths and is packaged 30 capsules per bottle. For the 12 mg dose, you will be required to take three 4 mg capsules for a total of 12 mg once a day. For the 8 mg dose, you will be required to take two 4 mg capsules for a total of 8 mg once a day.

  If, during the study, you experience any side effects for lenvatinib, the dose may be temporarily stopped and/or the dose reduced. The study staff will provide you with enough supply of study drug to last for approximately one month. At the start of each cycle, you will return any unused capsules and bottles to the clinic. Study drug should be taken at the same time each day, preferably in the morning with or without food. If you vomit after taking your dose you should not take another dose until your next day, and take the dose at the same time you normally would. Grapefruit (juice) and star fruit must not be taken during the study as these fruits may interfere with the blood levels of study drug. The study staff will provide you with additional dosing information.

- Sorafenib comes in 200 mg tablets. If you are taking sorafenib made in Europe, the package will be a box of 112 tablets. If you are taking sorafenib made in the United States, the package will be a bottle of 120 tablets.

  If during the study, you experience side effects for sorafenib, the dose may be temporarily stopped and/or the dose reduced per the instructions on the sorafenib drug label. These instructions may be different depending on which country you are in.

If you decide to join and qualify for this study, you will follow the schedule below and have the following procedures done.

In this study, every treatment “Cycle” is 28 days in duration.

Study Procedures and Schedule

- Randomization or assignment to either lenvatinib or sorafenib. This is discussed further in the above section entitled "Assignment into Your Study Group"
- Vital signs: blood pressure, heart rate, temperature, breathing rate including weight
- Questionnaires to test your general health and quality of life called EORTC-QLQ-C30, EORTC-QLQ-HCC18 and EQ-5D
- If you are taking lenvatinib, two blood samples to find out the amount of study drug in your blood will be drawn: 30 minutes to 4 hours after your dose and again, 6 to 10 hours after you have taken the dose. (A total of 12 mL of blood, which is equivalent to approximately 2.5 teaspoons, will be taken)
- You will also be asked about other medications you are taking and if you have any side effects from the study drug

Cycle 1, Day 8:

- You will be contacted by phone on Day 8 and asked if you have experienced any side effects. An unscheduled visit may occur prior to Cycle 1, Day 15 if deemed necessary by your study doctor.

- You will also be asked about other medications you are taking and if you have any side effects from the study drug

Cycle 1, Day 15:

- Vital signs: blood pressure, heart rate, temperature, and breathing rate including weight
- Complete physical examination
- Blood tests (total of about 1-2 teaspoons or 5 -10 mL of blood will be taken):
    - Chemistry (test that look at your kidney, liver, thyroid function and blood salts),
    - Hematology (test looking at the amount of red blood cells – cells that carry oxygen, white blood cells – cells that fight infection, and platelets – cells that help you form clots, in your blood)
- Urine test to check for any protein or sugar
- If you are taking lenvatinib,two blood samples to find out the amount of study drug in your blood will be drawn: before your first dose and 2-12 hours after your dose. (A total of 12 mL of blood, which is equivalent to approximately 2.5 teaspoons, will be taken)
- You will also be asked about other medications you are taking and if you have any side effects from the study drug

Cycle 2, Day 1:

- Assessment of your ability to do daily activities
- Vital signs: blood pressure, heart rate, temperature, breathing rate including weight
- Complete physical examination
- Heart Test:
    - Electrocardiogram (ECG) - An ECG records the rhythms and electrical activity of your heart. A number of electrodes (small, sticky patches) are stuck on your arms,

legs and chest. The electrodes are connected to a machine that records the electrical signals of each heartbeat and monitors how your heart is working

- Blood tests (total of about 1-2 teaspoons or 5 -10 mL of blood will be taken):
  - Chemistry (test that look at your kidney, liver, thyroid function and blood salts),
  - Hematology (test looking at the amount of red blood cells – cells that carry oxygen, white blood cells – cells that fight infection, and platelets – cells that help you form clots, in your blood)
- Urine test to check for any protein or sugar
- Test of liver cirrhosis, result of liver disease that could lead to loss of liver function. (Child-Pugh score test)
- Questionnaires to test your general health and quality of life called EORTC-QLQ-C30, EORTC-QLQ-HCC18 and EQ-5D
- Blood clotting exam to test for bleeding problems (2.7 mL or less than 1 teaspoon of blood will be taken)
- Special chemistry test to check for liver and thyroid gland problems (6.7 mL or about 1 and ½ teaspoons of blood will be taken)
- If you are taking lenvatinib, three blood samples to find out the amount of study drug in your blood will be drawn: before your first dose, 30 minutes to 4 hours after your dose and again, 6 to 10 hours after you have taken the dose. (A total of 18 mL of blood, which is equivalent to approximately 3.5 teaspoons, will be taken)
- You will be asked about other medications you are taking and if you have any side effects from the study drug

<u>Cycle 2, Day 15:</u>

- Vital signs: blood pressure, heart rate, temperature, breathing rate including weight
- Blood tests (total of about 1-2 teaspoons or 5 -10 mL of blood will be taken):
  - Chemistry (test that look at your kidney, liver, thyroid function and blood salts),
  - Hematology (test looking at the amount of red blood cells – cells that carry oxygen, white blood cells – cells that fight infection, and platelets – cells that help you form clots, in your blood)
- Urine test to check for any protein or sugar
- You will be asked about other medications you are taking and if you have any side effects from the study drug

<u>Cycle 3 Onwards</u>:

You will have the following tests performed:

<u>Day 1 of each cycle</u>:

- Assessment of your ability to do daily activities
- Vital signs: blood pressure, heart rate, temperature, breathing rate including weight
- Complete physical examination
- Heart Test:
  - Electrocardiogram (ECG) - An ECG records the rhythms and electrical activity of your heart. A number of electrodes (small, sticky patches) are stuck on your arms,

legs and chest. The electrodes are connected to a machine that records the electrical signals of each heartbeat and monitors how your heart is working

- Blood tests (total of about 1-2 teaspoons or 5 -10 mL of blood will be taken):
    - Chemistry (test that look at your kidney, liver, thyroid function and blood salts),
    - Hematology (test looking at the amount of red blood cells – cells that carry oxygen, white blood cells – cells that fight infection, and platelets – cells that help you form clots, in your blood)
- Urine test to check for any protein or sugar
- Test of liver cirrhosis, result of liver disease that could lead to loss of liver function. (Child-Pugh score test)
- Questionnaires to test your general health and quality of life called EORTC-QLQ-C30, EORTC-QLQ-HCC18 and EQ-5D
- Blood clotting exam to test for bleeding problems (2.7 mL or less than 1 teaspoon of blood will be taken)
- Special chemistry test to check for liver and thyroid gland problems (6.7 mL or about 1 and ½ teaspoons of blood will be taken)
- On cycles 3, 4, 5 & 6, if you are taking lenvatinib, a blood sample to find out the amount of study drug in your blood will be drawn before you dose. (A total of 6 mL of blood, which is equivalent to approximately 1.5 teaspoons will be taken)You will be asked about other medications you are taking and if you have any side effects from the study drug

Day 15 of each cycle (or more frequently depending on your condition):

- Vital signs: blood pressure, heart rate, temperature, breathing rate including weight
- Urine test to check for any protein or sugar
- You will be asked about other medications you are taking and if you have any side effects from the study drug

All participants in the study:

Should you experience any side effects during the study, you may need to return to the clinic more often until the side effects resolve and it is determined to be safe for you to continue on study. Additional blood may be taken during the start of a side effect and again once it resolves.

Additional tests:

- 24-hour urine protein tests – for patients with high levels of protein detected on the urine tests (See "What are the Potential Risks and Discomforts if I Choose to Take Part in this Study?" Section), a 24-hour urine collection will be done (you will collect all the urine passed during this period in a container provided to you by the study staff) to determine the amount of protein excreted into the urine by the kidneys. The results of this test will help guide your treatment. If the results of this test show that you have high levels of protein in your urine, study drug may be temporarily stopped and/or the dose reduced.
- Additional blood may be taken to check your liver if you have a history of bone metastases.

Measurements of your tumor sites or "tumor response evaluation" to see how your cancer is responding:

- Every 8 weeks: tumor assessment of your cancer; CT scan(s) and/or MRI(s), of chest, abdomen, pelvis and other areas if needed
- CT or MRI of the brain will be performed if your doctor feels it is needed

These tests may be done more frequently, if necessary

End of Study Visit

If you do not wish to continue in the study or if you feel the study drug is not benefiting you, or your doctor decides you should not continue in the study, the following end of study procedures will be carried out for your safety within 30 days after your last dose.

- Assessment of your ability to do daily activities
- Vital signs: blood pressure, heart rate, temperature, breathing rate including weight
- Complete physical examination
- Heart Tests:
  - Electrocardiogram (ECG) - An ECG records the rhythms and electrical activity of your heart. A number of electrodes (small, sticky patches) are stuck on your arms, legs and chest. The electrodes are connected to a machine that records the electrical signals of each heartbeat and monitors how your heart is working.
  - Echocardiogram or MUGA Scan - An echocardiogram is an ultrasound scan of the heart. Ultrasound is a very high frequency sound that you cannot hear, but it can be emitted and detected by special machines and is used to build up a detailed picture of the heart. You will be lying down on a bed and a small doppler (with gel on it) will reflect pictures onto a screen of how your heart is pumping. This test gives good information about the structure and function of your heart. A MUGA Scan (Multi Gated Acquisition Scan) is a nuclear medicine test used to evaluate the function of the heart ventricles (pumping chambers). The MUGA test involves the injection of a radioactive marker into the bloodstream. A scanner is then used to provide a movie-like image of the beating heart, which allows the doctor to determine the health of the heart's major pumping

- Blood tests (total of about 1-2 teaspoons or5 -10 mL of blood will be taken):
  - Chemistry (test that look at your kidney, liver, thyroid function and blood salts)
  - Hematology (test looking at the amount of red blood cells – cells that carry oxygen, white blood cells – cells that fight infection, and platelets – cells that help you form clots, in your blood)
  - Urine or blood pregnancy test (if you are a woman who is able to become pregnant)
- Urine test to for any protein or sugar
- Test of liver cirrhosis, result of liver disease that could lead to loss of liver function. (Child-Pugh score test)
- Questionnaires to test your general health and quality of life called EORTC-QLQ-C30, EORTC-QLQ-HCC18 and EQ-5D
- Blood clotting exam to test for bleeding problems (2.7 mL or less than 1 teaspoon of blood will be taken)
- Special chemistry test to check for liver and thyroid gland problems (6.7 mL or about 1 and ½ teaspoons of blood will be taken)

- Tumor response evaluation – Using CT scan(s), MRI(s), x-ray(s), unless done in the previous 4 weeks.
- You will be asked to report any symptoms that you have experienced since the last visit, whether they are related to the drug or not. You will also be asked to report all medications you are taking.

Follow Up

If you have stopped participating in the study and you have not had a diagnosis of progressive disease (your tumor has increased in size or you have new lesions not previously seen), you will still be asked to come to the clinic for follow up every 8 weeks to have a tumor response evaluation. These tumor response evaluations will stop when you start treatment with another anti-cancer treatment or if you are diagnosed with progression of your disease.

You will be followed by telephone or clinic visit every 12 weeks when you stop taking study medication.

Extension Phase Once Primary Analysis is Complete

When the study is fully enrolled and the primary analysis is complete (your study doctor will inform you on when this has occurred), all subjects still receiving lenvatinib or sorafenib will continue taking medication and enter the extension phase of the study.

Extension Phase Tests and Procedures

You will have the following tests and procedures performed during the extension phase:

Day 1of each cycle

- Assessment of your ability to do daily activities
- Complete physical exam including, weight and vital signs (blood pressure, heart rate, temperature, and breathing rate)
- Heart Test:
  - Electrocardiogram (ECG) - An ECG records the rhythms and electrical activity of your heart. A number of electrodes (small, sticky patches) are stuck on your arms, legs and chest. The electrodes are connected to a machine that records the electrical signals of each heartbeat and monitors how your heart is working
- Blood tests (total of about 1-2 teaspoons or 5 -10 mL of blood will be taken):
  - Chemistry (test that look at your kidney, liver, thyroid function and blood salts),
  - Hematology (test looking at the amount of red blood cells – cells that carry oxygen, white blood cells – cells that fight infection, and platelets – cells that help you form clots, in your blood)
- Urine test to check for any protein or sugar
- Test of liver cirrhosis, result of liver disease that could lead to loss of liver function. (Child-Pugh score test)
- Questionnaires to test your general health and quality of life called EORTC-QLQ-C30, EORTC-QLQ-HCC18 and EQ-5D
- Blood clotting exam to test for bleeding problems (2.7 mL or less than 1 teaspoon of blood will be taken)

- Special chemistry test to check for liver and thyroid gland problems (6.7 mL or about 1 and ½ teaspoons of blood will be taken)
- You will also be asked about other medications you are taking and if you have any side effects from the study drug

Day 15 of each cycle

- Vital signs: blood pressure, heart rate, temperature, breathing rate including weight
- Urine test to check for any protein or sugar
- You will also be asked about other medications you are taking and if you have any side effects from the study drug

All participants in the extension phase:

Should you experience any side effects during the study, you may need to return to the clinic more often until the side effects resolve and it is determined to be safe for you to continue on study. Additional blood may be taken during the start of a side effect and again once it resolves.

Additional tests:

- 24-hour urine protein tests – for patients with high levels of protein detected on the urine tests (See "What are the Potential Risks and Discomforts if I Choose to Take Part in this Study?" Section), a 24-hour urine collection will be done (you will collect all the urine passed during this period in a container provided to you by the study staff) to determine the amount of protein excreted into the urine by the kidneys. The results of this test will help guide your treatment. If the results of this test show that you have high levels of protein in your urine, study drug may be temporarily stopped and/or the dose reduced.
- Additional blood may be taken to check your liver if you have a history of bone metastases.

Measurements of your tumor sites or "tumor response evaluation" to see how your cancer is responding:

- Every 8 weeks: tumor assessment of your cancer; CT scan(s) and/or MRI(s), chest, abdomen, pelvis and other areas if needed
- CT or MRI of the brain will be performed if your doctor feels it is needed

These tests may be done more frequently, if necessary.

End of Study Visit

If you do not wish to continue in the study, or if the study drug is not benefiting you; or your doctor decides you should not continue in the study, the following procedures will be carried out for your safety within 30 days after your last dose:

- Assessment of your ability to do daily activities
- Vital signs: blood pressure, heart rate, temperature, breathing rate including weight
- Complete physical examination

- Heart Tests:
    - Electrocardiogram (ECG) - An ECG records the rhythms and electrical activity of your heart. A number of electrodes (small, sticky patches) are stuck on your arms, legs and chest. The electrodes are connected to a machine that records the electrical signals of each heartbeat and monitors how your heart is working
    - Echocardiogram or MUGA Scan - An echocardiogram is an ultrasound scan of the heart. Ultrasound is a very high frequency sound that you cannot hear, but it can be emitted and detected by special machines and is used to build up a detailed picture of the heart. You will be lying down on a bed and a small doppler (with gel on it) will reflect pictures onto a screen of how your heart is pumping. This test gives good information about the structure and function of your heart. A MUGA Scan (Multi Gated Acquisition Scan) is a nuclear medicine test used to evaluate the function of the heart ventricles (pumping chambers). The MUGA test involves the injection of a radioactive marker into the bloodstream. A scanner is then used to provide a movie-like image of the beating heart, which allows the doctor to determine the health of the heart's major pumping chambers
- Blood tests (total of about 1-2 teaspoons or5 -10 mL of blood will be taken):
    - Chemistry (test that look at your kidney, liver, thyroid function and blood salts)
    - Hematology (test looking at the amount of red blood cells – cells that carry oxygen, white blood cells – cells that fight infection, and platelets – cells that help you form clots, in your blood)
    - Urine or blood pregnancy test (if you are a woman who is able to become pregnant)
- Urine test to check for any protein or sugar
- Test of liver cirrhosis, result of liver disease that could lead to loss of liver function. (Child-Pugh score test)
- Questionnaires to test your general health and quality of life called EORTC-QLQ-C30, EORTC-QLQ-HCC18 and EQ-5D
- Blood clotting exam to test for bleeding problems (2.7 mL or less than 1 teaspoon of blood will be taken)
- Special chemistry test to check for liver and thyroid gland problems (6.7 mL or about 1 and ½ teaspoons of blood will be taken)
- Tumor response evaluation – Using CT scan(s), MRI(s), x-ray(s), unless done in the previous 4 weeks.
- You will be asked to report any symptoms that you have experienced since the last visit, whether they are related to the drug or not. You will also be asked to report all medications you are taking.

Follow Up

You will be followed for survival by telephone or clinic visit every 12 weeks when you stop taking study medication. The Sponsor of the study, Eisai, may stop this follow-up after they have analyzed the primary results of the study.

What Are My Responsibilities While I am in the Study?

You must be honest and complete in giving your medical history. This history also includes any medicines that you have taken, or are taking now. It also includes any other clinical research studies that you have taken part in or are in now.

It is important that you follow the instructions of the study doctor and study team. It is important that you come to all of your scheduled study visits. It is also important that you follow the schedule when you take the study drug.

It is important that during the study, you tell the study doctor and/or the study team all changes in how you feel. You need to tell them even if you do not believe these changes are related to the study. This may include mental or emotional changes. You will be checked during the study for side effects. You will also be checked for other injuries or illnesses that may happen while you are in the study.

You cannot take certain medicines during the research study. This may include foods or supplements. Before taking any medicines besides the study drug, you must first ask the study doctor. These medicines may be ones that are ordered by a doctor. They also could be drugs like allergy medicines, cough and cold medicines and pain relievers. They may also be vitamins, herbs and minerals. Grapefruit juice should not be drunk, nor grapefruit or starfruit eaten during the study.

Only you, the study subject, can take the study drug. Because the Sponsor of this study owns the study drug, all unused study drug must be returned to the study doctor. This includes any empty drug containers.

<u>What are the Potential Risks and Discomforts if I Choose to Take Part in this Study?</u>

In any research study, there may be side effects, complications, and/or injury that are both expected and unexpected. Such reactions, which may lead to serious injury or death, could occur through no fault of your own, the study staff, or the study sponsor.

Lenvatinib is approved in the US, Europe and Japan for use in the treatment of progressive or advanced thyroid cancer in adults when radioactive iodine treatment has not helped to stop the disease. Lenvatinib is also being tested for the treatment of other types of cancer and in this setting is considered to be an investigational drug. Lenvatinib has been given to approximately 2000 subjects in research studies. It is not possible to predict all of the risks and unwanted effects that might happen if you are given lenvatinib either alone or in combination with other drugs. It is possible that new side effects not described here may occur in this study.

Lenvatinib belongs to a type of anti-cancer treatment known as receptor tyrosine kinase inhibitors (RTKIs). Tyrosine kinases are proteins which are involved in the growth of cells and the development of new blood vessels that supply themand can be present in high amounts in cancer cells, By blocking their action on cell receptors lenvatinib may slow the rate at which the cancer cells grow and help to cut off the blood supply that feeds the cancer.

The known side effects of lenvatinib are listed below. Throughout the study, your study doctor will monitor your symptoms, blood pressure readings, and laboratory tests. If you develop any side effects your study doctor may need to temporarily stop and/or change the dose of your study drug.

Possible side effects

Like all medicines, this medicine can cause side effects, although not everybody will get all or even some of the side effects listed. The following side effects may happen with this medicine.

Tell your study doctor straight away if you notice any of the following side effects because you may need urgent medical treatment:

- feeling numb or weak on one side of your body, severe headache, seizure, confusion, difficulty talking, vision changes or feeling dizzy - these may be signs of a stroke, bleeding on your brain, or the effect on your brain of a severe increase in blood pressure
- swelling of the calf often associated with warmth or tenderness, sudden onset of shortness of breath, rapid breathing, tightening of chest or chest pain, cough or coughing up blood, rapid heart rate and a blue tinge to the lips – these may be signs of a blood clot in your legs or your lungs
- chest pain or pressure, pain in your arms, back, neck or jaw, being short of breath, rapid or irregular heart rate, coughing, bluish colour to lips or fingers, feeling very tired – these may be signs of a heart problem
- severe pain in your belly (abdomen) - this may be due to a hole in the wall of your gut or a fistula (a hole in your gut which links through a tube-like passage to another part of your body or skin).
- black, tarry, or bloody stools, or coughing up of blood - these may be signs of bleeding inside your body
- diarrhoea, feeling and being sick - these are very common side effects that can become serious if they cause you to become dehydrated, which can lead to kidney failure. Your doctor can give you medicine to reduce these side effects.

Very common (may affect more than 1 in 10 people)

- high blood pressure
- loss of appetite or weight loss
- feeling sick and being sick, constipation, diarrhoea, abdominal pain, indigestion
- feeling very tired or weak
- hoarse voice
- cough
- swelling of the legs
- rash
- dry, sore, or inflamed mouth, odd taste sensation
- swelling and inflammation of the joints; and stiff muscles, bones and joints
- feeling dizzy
- hair loss
- bleeding (most commonly nose bleeds, but may include bleeding from other sites such as blood in the urine, bruising, bleeding from the gums or gut wall)
- trouble sleeping

- changes in urine tests for protein (high)
- urinary infections (increased frequency in urination and pain in passing urine)
- pain – muscle, joint, headache, back
- redness, soreness and swelling of the skin on the hands and feet (hand-foot syndrome)
- changes in blood test results for potassium levels (low) and calcium levels (low) – may increase the chance of having an abnormal heart rhythm
- changes in blood test results for cholesterol (high) and thyroid stimulating hormone (high)
- bruising and difficulty in wound healing - signs of low level of platelets in the blood
- changes in blood test results for white blood cells (low) which may increase risk of infections
- underactive thyroid (may result in fatigue, weakness, dry skin, hair loss, intolerance to cold)

Common (may affect up to 1 in 10 people)

- low blood pressure
- loss of body fluids (dehydration)
- heart attack or abnormal heart rhythm
- dry skin, thickening and itching of the skin, redness of palm of the hands
- feeling bloated or having gas in the bowel
- heart problems or blood clots in the lungs (difficulty breathing, chest pain) or other organs
- feeling unwell
- stroke
- anal fistula (a small channel that forms between the anus and the surrounding skin which may require surgery to repair)
- changes in blood test results for liver, kidney, blood magnesium (low), and blood protein level (low), kidney failure (usually a result of dehydration from diarrhea and vomiting, and reversible and treatable with intravenous fluids and/or good hydration)

Uncommon (may affect up to 1 in 100 people)

- painful infection or irritation near the anus
- mini-stroke
- liver damage
- decreased pumping ability of the heart
- splenic infarction (severe pain in the upper left part of the belly (abdomen) which may be associated with fever, chills, nausea and vomiting)
- posterior reversible encephalopathy syndrome (PRES) a condition that may have the following symptoms: headache, confusion, convulsions and vision disturbance. An MRI scan may be required to diagnose this condition.

If any new information about lenvatinib is discovered during the course of the study that may impact your safety or willingness to participate in the study you will be notified by the study doctor. It is possible that you could have side effects of lenvatinib that nobody knows about. You

should get medical help and contact the study doctor or nurse if you have any of these or any other side effects during the study. It is important to inform the study doctor or nurse about any symptoms you experience, as they may be able to prescribe medications to treat these. Please tell them if you have any problems with your health or the way you feel during the study, whether or not you think they are related to the study drug.

Risks to Pregnant Women

Contraception, pregnancy and breastfeeding

- If you could become pregnant, use highly effective contraception while takingthe study drug, and for at least one month after your last dose .Do not take lenvatinib if you are planning to become pregnant during the study. This is because it may seriously harm your baby.
- If you become pregnant while taking lenvatinib, tell your study doctor immediately. Your study doctor will help you decide whether the study drug should be continued.
- Do not breastfeed if you are taking lenvatinib. This is because the study drug may seriously harm your baby.

Sorafenib

Sorafenib (also known as Nexavar®) is a *multikinase inhibitor*. It works by slowing down the rate of growth of cancer cells and cutting off the blood supply that keeps cancer cells growing.

Sorafenib is a drug approved by the FDA and other health authorities for the treatment of advanced kidney cancer when standard therapy has not helped to stop the disease or is considered unsuitable, differentiated thyroid cancer and for advanced liver cancer.

Below lists the more commonly seen side effects of the sorafenib:

Very common: may affect more than 1 in 10 users

- Diarrhoea
- Feeling sick (*nausea*)
- Feeling weak or tired
- Pain (including mouth pain, abdominal pain, headache, bone pain, tumour pain)
- Hair loss
- Flushed or painful palms or soles (hand foot syndrome)
- Itching or rash
- Throwing up (*vomiting*)
- Bleeding (including bleeding in the brain, gut wall and respiratory tract; *haemorrhage*)
- High blood pressure, or increases in blood pressure
- Infections
- Loss of appetite *(anorexia)*
- Constipation
- Joint pain *(arthralgia)*
- Fever
- Weight loss
- Dry skin

Common: may affect up to 1 in 10 users

- Flu-like illness
- Indigestion *(dyspepsia)*
- Difficulty swallowing *(dysphagia)*
- Inflamed or dry mouth, tongue pain *(stomatitis and mucosal inflammation)*
- Low calcium levels in the blood *(hypocalcaemia)*
- Low potassium levels in the blood (*hypokalaemia*)
- Muscle pain (*myalgia*)
- Disturbed sensations in fingers and toes, including tingling or numbness *(peripheral sensory*
- *neuropathy)*
- Depression
- Erection problems (*impotence*)
- Altered voice *(dysphonia)*
- Acne
- Inflamed, dry or scaly skin that sheds *(dermatitis, skin desquamation)*
- Heart failure
- Heart attack (*myocardial infarction*) or chest pain
- Tinnitus *(ringing sound in the ear)*
- Kidney failure
- Abnormally high levels of protein in the urine (*proteinuria*)
- General weakness or loss of strength *(asthenia)*
- Decrease in the number of white blood cells *(leucopenia and neutropenia)*
- Decrease in the number of red blood cells *(anaemia)*
- Low number of platelets in the blood (*thrombocytopenia*)
- Inflammation of hair follicles *(folliculitis)*
- Underactive thyroid gland *(hypothyroidism)*
- Low sodium levels in the blood *(hyponatraemia)*
- Distortion of the sense of taste *(dysgeusia)*
- Red in the face and often other areas of the skin *(flushing)*
- Runny nose *(rhinorrhoea)*
- Heartburn *(gastro oesophageal reflux disease)*
- Skin cancer *(keratoacanthomas/squamous cell cancer of the skin)*
- A thickening of the outer layer of the skin *(hyperkeratosis)*
- A sudden, involuntary contraction of a muscle *(muscle spasms)*

Uncommon side effects for sorafenib which were previously seen in less than 1 in 100 subjects are not listed here but can be provided by your study doctor. Please talk to your study doctor if you have any questions about side effects.

It is important to inform the study doctor or nurse about any symptoms you experience, as they may be able to prescribe medications to treat these. Please tell them if you have any problems with your health or the way you feel during the study, whether or not you think they are related to the study drug.

Some of the events listed above were considered to be serious and were fatal. Other side effects which occurred less frequently were also considered to be serious and have in some instances also resulted in death. These have included:

- Gastrointestinal perforation (a hole developing in the wall of the gut)
- Liver injury (drug-induced hepatitis, which may have the following symptoms: chills, fever, rash, itching, jaundice, joint pains, headache, abdominal pain, loss of appetite, nausea and vomiting).
- Prolongation of 'QT interval' on an electrocardiogram (heart tracing) - this can lead to abnormal heart rhythms

- Pneumonitis (inflammation of lung tissue) – this can be severe and potentially life-threatening
- Toxic epidermal necrolysis and Steven's Johnson syndrome, life threatening skin conditions where the top layer of skin may detach leaving the body at risk of severe and widespread infection (sepsis)
- Posterior reversible encephalopathy syndrome (PRES) - a condition that may have the following symptoms: headache, confusion, convulsions and vision disturbance.
    - MRI is necessary to confirm the diagnosis of PRES
- Pancreatitis (inflammation of the pancreas, which is a small gland at the back of the abdomen)
- Cholecystitis (inflamed gall bladder)
- Anaphylactic reaction (an allergic reaction which may have the following symptoms: difficulty breathing, rash , raised bumps or hives, itching and swelling of the face and throat)

- **Risk of Fetal Harm**
  There are no adequate and well-controlled studies in pregnant women using NEXAVAR. However, based on its mechanism of action and findings in animals, NEXAVAR may cause fetal harm when administered to a pregnant woman. Sorafenib caused embryo-fetal toxicities in animals at maternal exposures that were significantly lower than the human exposures at the recommended dose of 400 mg twice daily. Advise women of childbearing potential to avoid becoming pregnant while on NEXAVAR because of the potential hazard to the fetus.

Frequency of event listed below is not known and cannot be estimated from the available data.

- Impaired brain function that can be associated with e.g. drowsiness, behavioural changes, or confusion (encephalopathy).

<u>Risks to Pregnant Women</u>

If you are a woman and it is possible for you to become pregnant, a pregnancy test will be performed at study entry and you must confirm that you have not had unprotected sex within 30 days of entering the study. If it is possible for you to become pregnant, you must agree to use a highly effective method of contraception throughout the entire study period and for 30 days after the study drug is stopped. Examples of highly effective methods of contraception are: total abstinence, an intrauterine device (IUD) or intrauterine system (IUS), a double-barrier method (condom and diaphragm with spermicide foam, gel, cream etc.), hormonal contraceptives such as an oral contraceptive or a contraceptive implant, or a partner who has had a vasectomy with no measurable level of sperm in the semen. If you are using hormonal contraceptives, you must have been on a stable dose of the same product for at least 4 weeks before taking the study medication and you must continue to use the same contraceptive throughout the entire study period and for 30 days after study drug discontinuation. If you are currently abstinent, you must agree to use a double barrier method of contraception as described above if you become sexually active during the study.

You will be exempt from these contraceptive requirements if you are unable to become pregnant i.e.:

- Women who have had a hysterectomy (uterus removed), bilateral oophorectomy (both ovaries removed), bilateral tubal ligation and documentation of the procedure at least one month before the start of the study.

- Postmenopausal women in the appropriate age group who had their last period more than one year before start of the study treatment.

If you are a man with a partner who is able to become pregnant, you and/ or your partner must use a highly effective method of contraception as described above, from at least the first day of your partner's last normal menstrual period, throughout the entire study period, and for 30 days after stopping the study drug. Those with partners using hormonal contraceptives must also be using an additional approved method of contraception.

If you or your partner becomes pregnant during the study, there may be risks to both you and your unborn child, and these risks are not known. Therefore, if you think you might be pregnant, you must notify the study doctor immediately.

What are the other possible risks and discomforts?

Blood drawing: Local pain, bruising, bleeding, blood clot formation, and, in rare instances, an infection might occur in the area where blood is drawn. There is also the possibility of dizziness or fainting while your blood is being drawn. [The decision to use a catheter (a thin tube) for blood collecting is made by the study staff. The study staff will explain the catheter to you if its use is necessary.]

The following radiological procedures to evaluate the effect of lenvatinib on your tumor:

CT scan(s) (computerized tomography): This is like an x-ray machine that uses a computer to take pictures of the organs of the body. Your actual CT scan will only take a few minutes. You may feel "closed in" from being in the enclosed space while having the CT scan. However, the scanner is open at both ends. There is also an intercom that allows you to talk to the staff if you feel anxious. If a CT of your belly or hip area is being done, you will usually be asked to drink liquid about 60-90 minutes before the CT scan. This liquid will help your stomach and bowel to be seen better in the CT pictures. This liquid may sometimes cause nausea or vomiting. Right before the CT scan, you will also usually be given an iodinated CT contrast agent (dye) in your vein with a small plastic tube. This dye helps to provide better CT pictures of your organs and tumor(s). The contrast dye used for a CT scan may cause flushing, nausea or vomiting. It also can very rarely cause a severe allergic reaction which can be treated but which may be life-threatening. The contrast dye may also rarely cause kidney damage. This is more likely if you are dehydrated or have diabetes.

MRI Scans: The MRI uses magnetic waves to look at soft tissues of the body. There are times when you may have MRI scans instead of CT scans done to evaluate your tumor in certain locations. MRI scanners use a large magnet and radio waves to take pictures of your body. The scanning takes about 30 - 60 minutes depending on what part of your body is scanned. The effects of the magnetic fields in a MRI scanner have been widely studied. There are no known risks from being exposed to the magnetic fields. Before each scan, you will be asked a series of questions by the MRI staff to be sure that you do not have any medical reasons that stop you from having an MRI. You should not have a MRI if you have a pacemaker, metallic cardiac valve(s), or certain types of metallic aneurysm clips. You should not have a MRI if you have implanted, electronic infusion pumps or other metallic pieces in your body (bullets, shrapnel fragments). You will be asked to change into a gown and remove all metallic objects for scanning. You will lie on a flat table that will move into a horizontal tube that is within a large magnet. In order to lower the loud banging noises from the machine during the scanning, you may wear headphones or put in

earplugs. You may feel “closed in” from being in the enclosed space while having the MRI scan. However, the scanner is open at both ends. There is an intercom so you can talk to the staff if you feel anxious. Some subjects cannot tolerate the MRI scan if they are very afraid of small spaces. For some MRI scans, you may get a MRI contrast material (a so-called gadolinium chelate or dye). This is given in your vein using a small needle or plastic tube. The MRI contrast material will be given in amounts that have been approved by Health Authorities You will not get the MRI contrast material if you have abnormal kidney function. It is uncommon, but you may feel local warmth/pain in the area where the needle was given. You may also have nausea/vomiting or headache. Serious allergic reactions that may be life threatening are very rare.

Risks of Contrast Material or Radioactivity Injection: You may have pain in the place where the needle was inserted. There may also be some swelling in that area. There is also a higher but very uncommon risk of infection where the needle was inserted. There is a small risk of an allergic reaction to these injections as described in the section on CT and bone scans, and MRI examinations.

Radiation Exposure: The number of CT scans you will require in this study (every 8 weeks) may be more than you would normally have if you were not on study. Your doctor may request that you have MUGA scans to check your heart function. If you take part in this study for one year, the total amount of radiation involved in having these CT and MUGA scans is equivalent to about 30-40 years' worth of natural background radiation. There is a risk with radiation exposure that a second cancer may develop, and you should discuss this risk with the study doctor.

The following procedures may be performed to evaluate any adverse effects on your heart. (You will either have a MUGA or ECHO scan, not both):

- Electrocardiogram (ECG): The ECG is a picture of the electrical action of the heart. During this procedure, you will need to lie still for a few minutes so electrodes are attached to your chest. The electrodes may cause some discomfort when they are put on and taken off your skin. If you are a male and have any chest hair, it may need to be shaved off on the areas where the electrodes will be placed.

- Echocardiogram or MUGA Scan - An echocardiogram is an ultrasound scan of the heart. Ultrasound is a very high frequency sound that you cannot hear, but it can be emitted and detected by special machines and is used to build up a detailed picture of the heart. You will be lying down on a bed and a small doppler (with gel on it) will reflect pictures onto a screen of how your heart is pumping. This test gives good information about the structure and function of your heart.
  A MUGA Scan (Multi Gated Acquisition Scan) is a nuclear medicine test used to evaluate the function of the heart ventricles (pumping chambers). The MUGA test involves the injection of a radioactive marker into the bloodstream. A scanner is then used to provide a movie-like image of the beating heart, which allows the doctor to determine the health of the heart’s major pumping chambers

Medications: Before taking any over the counter medicines, herbal products, vitamins or food supplements or any other types of special products for your health while participating in this study, please discuss this with the study doctor who will tell you whether it is appropriate to start or continue taking these medicines. You should also talk to the study doctor before taking any

medicines other doctors may have prescribed for you. This is to ensure that there are no harmful interactions between the study drug and any other medicines you are taking. Your study doctor may need to change or discontinue these for the duration of the study.

What are the Possible Benefits if I Take Part in this Study?

Taking part in this clinical study might not benefit you in any way. The study drug may not help you. Your condition may get better, get worse or stay the same. However, your taking part may give more scientific information about the study drug and/or unresectable hepatocellular carcinoma.

Will I be Paid to Take Part in this Study?

You will not be paid for taking part in this study.

What is the Source of Funding for this Study?

Funding for this study is provided by the Sponsor (Eisai). The research center will be paid by the Sponsor to conduct this study.

Will There be any Cost to Me to Take Part in this Study?

The study drug lenvatinib, and sorafenib will be provided by the Sponsor at no cost to you. Screening and any other tests or procedures necessary to determine your eligibility for participation in the study (as described above) will be paid by the sponsor. The sponsor will also pay for tests, procedures and drugs that are required as part of the study and not covered by your health insurance carrier, Medicare or any other third party payer. Many of the tests (blood tests, x-rays, urine test, CT/MRI scans) involved in this study are part of the standard evaluation and care for the treatment of your cancer. These standard tests may be covered by your health insurance carrier, Medicare or other third party payer and they will be billed for the costs of these tests that are covered by them in the same manner as if you were not part of this study. You will be billed for any additional costs your insurance carrier declines to pay, such as co-pays or deductibles. If you have any questions about medical and hospital charges, medical insurance coverage, or expenses related to the study, please discuss them with the study doctor and your insurance provider. Please include in your discussion the costs of treating side effects if that becomes necessary. Otherwise, you might have unexpected expenses from being in this study.

Is There Anything Else I Can Do for My Condition?

You may choose not to take part in this research study. The study doctor will discuss other treatments available to you for your cancer.

How Will I Know if There is Any New Information that Might Affect My Will to Continue in This Research Study?

You will be told of any new information learned during this study or any other study using lenvatinib that might make you change your mind about being in this study.

What if I Want to Stop Being in the Study?

You are free to stop being in the study at any time by notifying the study doctor.

If you want to stop receiving study medication and performing tests for the study but still agree to be followed to help monitor your health and condition, tell the study doctor or study staff. The study doctor or study staff may ask you some additional questions. These questions may include the reasons why you want to stop being in the study. If you, or the study doctor, decide to remove you from the study you will be asked to return all unused study drug. You will also be asked to return for follow-up visits to help monitor your heath and condition. You may be contacted by the study staff on a regular basis thereafter.

If you decide to stop taking part in the study and would not like to be further monitored for your health and condition, you must tell the study doctor in writing that you do not want us to collect more data about you. Even if you gave permission to collect more data, and then did not want more data collected, we may continue using the data we have already collected about you. This will help to maintain the reliability of the study. Other authorized parties, including the study sponsor, its consultants, contractors and agents may also continue to use the data. In addition, we are required to continue to report any results from the study related to the safety of the study drug to the Food and Drug Administration (FDA) and other Health Authorities.

If you choose not to participate in this study or withdraw for any other reason other than worsening of your cancer as confirmed by a central imaging laboratory, you will not be eligible to enroll in the extension phase of this study and receive lenvatinib.

What Will Happen if I am Injured During the Study?

If you participate in this study, you will be exposed to certain risks of injury and/or illness in addition to those connected with your condition. Please refer to the Section entitled "What are the Potential Risks and Discomforts if I Choose to Participate in this Study?

If you are injured or become ill as a result of being in this study, you should seek medical help immediately and arrange to notify the study doctor immediately.

- If you suffer an illness or injury that is caused by the study drug lenvatinib, or sorafenib or a proper study procedure, the Sponsor agrees to pay the medical expenses that are necessary to treat such illness or injury.

However, it is important that during the time you are in the study, you follow the directions of the study doctor/study staff. It is also important that during the time you are in the study, you follow the directions of this informed consent form. It is important that you tell the study doctor right away of your injury/illness.

Paying for your medical expenses does not mean that Eisai or anyone else is at fault or is liable.

No funds have been set aside by the Sponsor and there are no plans to pay you for any of the following costs.

- costs because of injuries due to your personal conduct outside of the study,
- costs for lost wages,
- costs for disability,
- costs for discomfort,
- costs for losses claimed by spouses or family members,
- costs for loss of companionship; and

- costs due to the treatment, progression or worsening of your disease, underlying condition or any other independent condition or disease.

It is up to you or your insurance company to pay for costs to treat any medical condition not caused by the study. You can get more information from the study doctor about medical treatment for injuries related to the study.

YOU DO NOT GIVE UP ANY OF YOUR LEGAL RIGHTS AS A RESEARCH SUBJECT OR YOUR RIGHT TO PURSUE A CLAIM THROUGH THE LEGAL SYSTEM BY SIGNING THIS CONSENT FORM.

Who Can I Contact About this Study or my Rights as a Research Participant?

If you have any study questions, you [or your caretaker] should call Insert Investigator Name[study doctor] at Insert Investigator Telephone #. After normal business hours you should call Insert After-Hours Telephone #.

If you have any injury or illness during the study you [or your caretaker] should call Insert Investigator Name[study doctor] at Insert Investigator Telephone #. After normal business hours you should call Insert After-Hours Telephone #.

If you [or your caregiver] have any questions about your rights as a research subject, you should call Insert IRB Name at Insert IRB Telephone #.

Will My Information Be Kept Confidential?

Your personal information will be kept confidential to the extent required by applicable law. This section explains how information collected about you for the study will be used. This information includes your medical history, procedures and test results. It also includes information about how you respond to treatments you get in the study. It also includes other medical information about you being in the study (together, "Study Information").

Study Information will be collected by the study doctor and/or research team and will be given to:

- the Sponsor (Eisai Inc.) and its affiliated Eisai companies.

- Sponsor's consultants who are helping the Sponsor conduct this study, including, contract research organizations and laboratories.

- the Institutional Review Boards (IRB) or Ethics Committees (committees that have reviewed this study to help monitor that the rights of the study participants are respected, and that the study is carried out in a safe and ethical manner).

The Sponsor may also give your study information to the FDA. The Sponsor may also give your study information to other governmental agencies. These agencies may be in other countries. This may be part of the process to try to get the Study Drug approved by the FDA and/or foreign government authorities. Study records may need to be "photocopied". Study records may be

collected by the Sponsor. Study records may be collected by the FDA. They may be collected by the IRB/IEC. They may be collected by US and International Regulatory agencies and boards.

In addition, if a medical emergency happens, your study information may be given to your doctor and emergency staff. Your full privacy cannot be promised. This is because of the need to give information to all of these parties. However, your confidentiality will be protected to the extent required by applicable laws and regulations. The results of this study may be shown in publications or at meetings, but you will not be identified by name. The results may be put in publications, but you will not be identified by name.

There are United States federal laws and regulations related to privacy. These laws can be found under the Health Insurance Portability and Accountability Act of 1996 ("HIPAA"). These laws apply to certain personal health information collected in connection with this study. Your permission to use this information is required. In addition to this Informed Consent Form, you will be asked to give your permission to use your personal health information. This is done by signing a separate HIPAA Authorization Form. Please ask the study site if you have any questions about the HIPAA Authorization Form.

A description of this clinical trial will be available on http://www.ClinicalTrials.gov, as required by U.S. Law. This Web site will not include information that can identify you. At most, the Web site will include a summary of the results. You can search this Web site at any time. The public will be able to read this information using the internet (your personal information will be kept confidential to the extent required by applicable law).

Research Participant Acknowledgement

- I have read and understand the information about the study and other information described in this form.
- I have had the opportunity to ask questions, which have been answered to my satisfaction.
- I understand that I will receive a signed and dated copy of this consent form.
- I understand that by signing this consent form, I have not waived any of the legal rights which I otherwise would have as a subject in a research study.
- This consent form is being voluntarily signed by me. I agree to take part in this study. I agree to the use and disclosure of the Study Information as described in this consent form.

- I understand I can stop the study at any time without penalty to me.

______________________________ ____________
Subject Name [Print or type] Date

______________________________ ____________
Witness Name: [Print or type] Date

INVESTIGATOR STATEMENT

I, the undersigned, certify that the subject signing this consent had the study fully and carefully explained to him/her by me and appears to reasonably understand the nature, risks, and potential benefits, if any, of his/her participation in this research study.

By:_______________________________ ___________
Study Doctor Name [Print or Type] Date

HIPAA Research Authorization
Authorization to use and Disclose Health Information

I agree to permit [Institution Name] and any of my doctors or other health care providers (together "Providers"), Principal Investigator and [his/her/their/its] collaborators and staff (together "Researchers"), to obtain, use and disclose health information about me as described below.

1. The health information that may be used and disclosed includes:

   - All information collected during the research and procedures described in the Informed Consent Form (the "Research"); and

   - Personal health information in my medical records that is relevant to the Research, which includes my past medical history, medical information from my primary care physician and other medical information relating to my participation in the study.

2. The Providers may disclose health information in my medical records to:

   - The Researchers;

   - Representatives of government agencies, review boards, and other persons who watch over the safety, effectiveness, and conduct of research; and

   - The sponsor of the Research, Eisai Inc. and its affiliates, agents and contractors assisting in the conduct or completion of the (together, "Sponsor").

3. The Researchers may use and share my health information:

   - Among themselves, with the Sponsor, and with the other participating Researchers to conduct the Research; and

   - As permitted by the Informed Consent Form.

4. The Sponsor may use and share my health information for purposes of the Research and as permitted by the Informed Consent Form.

5. Once my health information has been disclosed to a third party, federal privacy laws may no longer protect it from further disclosure.

6. Please note that:

   - You do not have to sign this Authorization, but if you do not, you may not participate in the Research. If you do not sign this Authorization, your right to other medical treatment will not be affected.

   - You may change your mind and revoke (take back) this Authorization at any time for any reason. To revoke this Authorization, you must write to the Research Study Personnel at:

NAME:
ADDRESS:
PHONE:

- However, if you revoke this Authorization, you will not be allowed to continue taking part in the Research. Also, even if you revoke this Authorization, the Providers, Researchers and the Sponsor may continue to use and disclose the information they have already collected to protect the integrity of the research or as permitted by the Informed Consent Form.

- While the Research is in process, you may not be allowed to see your health information that is created or collected by [Institution Name] during the course of the Research. After the Research is finished; however, you may see this information as described in [Institution Name], Notice of Privacy Practices.

7. This Authorization does not have an expiration (ending) date.

For California Sites:

The expiration date for this Authorization is 25 years after the study is completed.

8. You will be given a copy of this Authorization after you have signed it.

___

Signature of Participant Date Time

___

Printed Name of Participant

OR

___

Signature of Legally Authorized Representative Date Time

___

Printed Name of Legally Authorized Representative

___

Legally Authorized Representative Relationship to Study Subject

Eisai Protocol E7080-G000-304

A Multicenter, Randomized, Open-Label, Phase 3 Trial to Compare the Efficacy and Safety of Lenvatinib (E7080) Versus Sorafenib in First-Line Treatment of Subjects With Unresectable Hepatocellular Carcinoma

PHARMACOGENOMIC, BIOMARKER AND TISSUE SAMPLE INFORMATION SHEET AND CONSENT FORM

PURPOSE OF CONSENT

You are being invited to participate in research that will explore whether there may by a genetic or inherited reason for how subjects respond to medications. Your blood and tissue samples contain genes, which are made up of DNA and proteins which serve as the "instruction book" for the cells that make up our bodies. Your samples will help us study how genes interact with other factors to influence the development of diseases such as cancer Additionally, proteins found in your blood and tissue samples may be studied to help predict differences in response to treatment. Research on your blood and tissue samples may be performed to identify and characterize new diagnostic biomarkers, cells, proteins and/or nucleic acids related to cancer. Pharmacogenomic and biomarker research may help to understand how to diagnose and treat individuals with cancer and may facilitate development of new more effective therapies that will improve future medical care for cancer.

We will use the already collected (archived) tumor tissue from surgery or biopsy for research. A small amount of tumor DNA may be obtained from your tumor to understand what mutations have developed in the DNA to cause the cancer, as well as help scientists understand why your tumor may respond or not respond to the study treatment. The sponsor may examine genes they think may be important in your cancer, or response to the study drug. Because the development of cancer is complex, it is difficult to pre-specify all the genes that may be examined. Recently, technology has evolved that allows scientist to determine the entire DNA sequence of a genome for a reasonable cost. The sponsor may use this technology to understand how DNA taken from your tumor differs from DNA taken from your healthy blood cells or non-tumor cells obtained from your tumor biopsy.

The results of the Pharmacogenomic and Biomarker research may:

- Develop products or tests that may help patients do better on drugs or suffer fewer side effects from drugs. This research may help the Sponsor to develop a unique and predictive test (like a diagnostic test) that can help identify which patients may respond best to treatment and which patients may be more at risk for bad side effects from treatments.
- Find out how the study drug may work, and to better understand why the study drug may work well in some patients but not others and why some patient experience side effects while others do not.
- Find out more about what causes diseases and how to prevent and treat them. Many researchers use these samples to help develop new tests to diagnose diseases or develop new drugs to treat and possibly cure diseases.

PROCEDURES

You are being asked to donate the following samples for this research.

- One 6.0mL (about 1.5 teaspoons of blood) Genomic DNA sample at Cycle 1 Day 1

- One 6.0mL (about 1.5 teaspoons of blood) Serum biomarker sample at Cycle 1 Day 1 (predose), Cycle 1 Day 15, Day 1 of all following cycles, and the Off-Treatment Visit
- One 5.0mL (1 teaspoon of blood) Plasma biomarker sample at Cycle 1 Day 1 (predose), Cycle 1 Day 15, Day 1 of all following cycles, and the Off-Treatment Visit
- A sample of your tumor from a previous surgery or biopsy (a "historical or archive sample") will be collected if available and may be used to study your cancer if you have previously had tumor surgery (for example: endoscopy, laparoscopy, CT-guided biopsy or tumor surgery) for diagnosis or treatment of your cancer.

DISCLOSURE OF RESEARCH RESULTS

Many studies are exploratory research studies and may not yield information that will be clinically useful to patients for some time. Therefore, no information obtained from research studies will be given to you, your family or the medical staff. Research information from this sub-study will not become part of your medical records.

If there are research findings discovered while you are still actively participating in the main research study that will be critical safety findings for the patients enrolled in our clinical trial, the Sponsor may publish the results, contact all study doctors, and offer to pay for patients enrolled in the clinical trial to have additional testing performed. If important research findings are discovered after subjects have concluded participation in the clinical trial, the sponsor may publish results, present results in national meetings, and post results on our website in order to rapidly disseminate this information to doctors and patients.

To further protect your participation, information obtained from research studies will not be linked to your medical record and will be maintained separately from all clinical information obtained during the course of the main research study. Any data than can be used to uniquely identify you will not be included in the sample database.

FOR United States ONLY

There is a federal law called the Genetic Information Nondiscrimination Act (GINA). In general, this law makes it illegal for health insurance companies, group health plans, and most employers to discriminate against you based on your genetic information. However, it does not protect you against discrimination by companies that sell life insurance, disability insurance, or long-term care insurance.

POSSIBLE RISKS

Pharmacogenomic and biomarker samples will usually be collected with a scheduled blood draw for the main study, therefore, poses little additional risk. To further protect you, no genetic information will be returned to you or be put into your medical record.

Blood sampling may cause possible discomfort and pain, minor bruising, and swelling at the site of the needle stick

POSSIBLE BENEFITS TO THE STUDY VOLUNTEER

You will not receive any direct benefit as a result of taking part in this additional research. However, the knowledge gained from this research may benefit others.

ALTERNATIVES TO PARTICIPATING IN THIS STUDY

Since there will be no direct benefit to you by taking part in this research, the alternative is not to participate. You may still participate in E7080-G000-304 study if you choose not to participate in this sub-study.

COMPENSATION:
You will not be paid for donating your sample. Nor will there be any charge to you for your participation in this sub study.

COMPENSATION FOR INJURY:
If you are injured or become ill as a result of being in this pharmacogenomic or biomarker study, you should seek medical help immediately and arrange to notify the study doctor immediately.

If you suffer bodily injury during the study directly caused by a study procedure in accordance with the study protocol, the Sponsor, Eisai Inc., will pay your reasonable and necessary medical expenses for the treatment of such injury to the extent such injury is not attributable to any underlying or independently occurring condition or disease; provided that:

- The injury is solely and directly caused by the study procedure according to the protocol;
- You have followed the directions of the study doctor/staff and this informed consent form;
- The injury was not deliberately caused;
- Such expenses are not actually paid or reimbursed by medical, third party, or government insurance or programs; and
- You immediately notified the study doctor or any injury or illness and follow all directions you are given in regards to such injury.

No compensation has been made available to you for injuries resulting from your personal conduct or participation outside of the study protocol. Sponsor has no plans to pay you for any such expenses that have already been paid or reimbursed by your insurance or government programs, such as Medicare or Medicaid. Paying for your medical expenses does not mean that Eisai Inc. or anyone else is at fault or is liable.

There are no plans to pay you for any of the following costs.

- costs because of injuries due to your personal conduct outside of the study,
- costs for lost wages,
- costs for disability,
- costs for discomfort,
- costs for losses claimed by spouses or family members,
- costs for loss of companionship; and
- costs due to the treatment, progression or worsening of your disease, underlying condition or any other independent condition or disease.

It is up to you or your insurance company to pay for costs to treat any medical condition not caused by the study. You can get more information from the study doctor about medical treatment for injuries related to the study.

YOU DO NOT GIVE UP ANY OF YOUR LEGAL RIGHTS AS A RESEARCH SUBJECT OR YOUR RIGHT TO PURSUE A CLAIM THROUGH THE LEGAL SYSTEM BY SIGNING THIS CONSENT FORM.

CONFIDENTIALITY OF RECORDS:
Your blood and tissue samples will be coded with your subject number from the main study. This code number links your sample and results to your name. The Sponsor's researchers doing the tests will not know your identity from this code number. The Study Doctor will keep the list which links the code number to your name. The following people may access this list to make sure that the study is properly done.

- Employees of the FDA (Food and Drug Administration) or other government authorities,
- The Sponsor or its agents,

Your samples will be stored in a secure facility. Only authorized staff is allowed to enter the facility. Your samples may be transferred to other companies or to research partners working with the Sponsor. When your DNA or proteins are taken out of the blood samples a different unique number will be applied to your sample to further protect your genetic information, this is called Double Coding. The "key" that will link the sample with the number used on your blood sample taken at the clinic will be maintained in a secure system under strict supervision and security by authorized personnel and will not be released to clinicians or researchers.

Only your doctor will have the information that matches the code to your identifying information, such as your name, address, phone number, or social security number. Your doctor will keep the information that matches the code to this identifying information in a safeguarded database. Only very few, authorized people, who have specifically agreed to protect your identity, will have access to this database. All other researchers and personnel, including those who will be working with your samples and medical information, will not have access to any of the identifying information about you.

In addition, the Sponsor may share information from your samples with the U.S. Food and Drug Administration (FDA) and other government agencies in the United States and other countries. Due to the need to release your information to the agencies listed above, the Sponsor cannot promise you absolute privacy. However, the Sponsor will protect your privacy as much as possible under applicable laws and regulations. Your samples will not be sold, loaned or given to any other independent groups for their own use. Research partners working with the Sponsor are not allowed to share DNA samples with anyone else.

You will not be given the results of this research. The results will not be provided to any insurance company, your employer, your family or any of your doctors, nor will the results be included in your medical records. Your samples may be stored for testing for up to 15 years after the completion of this study, or until the sample is gone. When (or before) the 15-year period ends your sample(s) will be destroyed. Your samples may be stored for longer if a Heath Authority (or medicinal product approval agency) has active questions about the study. In this special circumstance, samples will be stored until Heath Authority (or medicinal product approval agency) questions have been addressed.

If any future testing is performed using your samples as described in this consent, no additional informed consent will be obtained from you and you will not be notified.

Taking part in this pharmacogenomic and biomarker research is completely voluntary. Whether or not you decide to participate in this research, you may still participate in the main study.

If, at any time, you should choose to withdraw from participation from this pharmacogenomic and biomarker research, all samples maintained in storage will be destroyed and all records linked to the samples will be deleted. However, data obtained from samples prior to withdrawal of consent will not be deleted.

SIGNIFICANT NEW DEVELOPMENTS

Any significant new developments during the course of this research that might influence your willingness to continue to take part will be reported to you through the study site.

Study Site:
Principal Investigator Name:
Address:
Phone:

QUESTIONS

You are encouraged to ask any questions related to this research. All of your questions should be answered to your satisfaction before you consent to participate in this study.

Research Participant Acknowledgement

- I have read and understand the information about the study and other information described in this form.
- I have had the opportunity to ask questions, which have been answered to my satisfaction.
- I understand that I will receive a signed and dated copy of this consent form.
- I understand that by signing this consent form, I have not waived any of the legal rights which I otherwise would have as a subject in a research study.
- This consent form is being voluntarily signed by me. I agree to take part in this study. I agree to the use and disclosure of the Study Information as described in this consent form.

- I understand I can stop the study at any time without penalty to me.

______________________________ ___________
Subject Name [Print or type] Date

______________________________ ___________
Witness Name: [Print or type] Date

16.1.3.3 Representative Written Information for Subjects

A Quality of Life Questionnaire was provided to the subjects during this study and is provided on the following pages. Only the most current version is provided, but the previous versions are available upon request.

- EORTC QLQ-C30 version 3
- EORTC QLQ-HCC18
- EQ-5D-3L

Printed copy from PhlexEview - Property of Eisai.

EORTC QLQ-C30 (version 3)

We are interested in some things about you and your health. Please answer all of the questions yourself by circling the number that best applies to you. There are no "right" or "wrong" answers. The information that you provide will remain strictly confidential.

Please fill in your initials: |_|_|_|_|
Your birthdate (Day, Month, Year): |_|_|_|_|_|_|_|_|
Today's date (Day, Month, Year): 31 |_|_|_|_|_|_|_|_|

		Not at All	A Little	Quite a Bit	Very Much
1.	Do you have any trouble doing strenuous activities, like carrying a heavy shopping bag or a suitcase?	1	2	3	4
2.	Do you have any trouble taking a long walk?	1	2	3	4
3.	Do you have any trouble taking a short walk outside of the house?	1	2	3	4
4.	Do you need to stay in bed or a chair during the day?	1	2	3	4
5.	Do you need help with eating, dressing, washing yourself or using the toilet?	1	2	3	4

During the past week:

		Not at All	A Little	Quite a Bit	Very Much
6.	Were you limited in doing either your work or other daily activities?	1	2	3	4
7.	Were you limited in pursuing your hobbies or other leisure time activities?	1	2	3	4
8.	Were you short of breath?	1	2	3	4
9.	Have you had pain?	1	2	3	4
10.	Did you need to rest?	1	2	3	4
11.	Have you had trouble sleeping?	1	2	3	4
12.	Have you felt weak?	1	2	3	4
13.	Have you lacked appetite?	1	2	3	4
14.	Have you felt nauseated?	1	2	3	4
15.	Have you vomited?	1	2	3	4
16.	Have you been constipated?	1	2	3	4

Please go on to the next page

Printed copy from PhlexEview - Property of Eisai.

**During the past week:**	**Not at All**	**A Little**	**Quite a Bit**	**Very Much**
17. Have you had diarrhea?	1	2	3	4
18. Were you tired?	1	2	3	4
19. Did pain interfere with your daily activities?	1	2	3	4
20. Have you had difficulty in concentrating on things, like reading a newspaper or watching television?	1	2	3	4
21. Did you feel tense?	1	2	3	4
22. Did you worry?	1	2	3	4
23. Did you feel irritable?	1	2	3	4
24. Did you feel depressed?	1	2	3	4
25. Have you had difficulty remembering things?	1	2	3	4
26. Has your physical condition or medical treatment interfered with your family life?	1	2	3	4
27. Has your physical condition or medical treatment interfered with your social activities?	1	2	3	4
28. Has your physical condition or medical treatment caused you financial difficulties?	1	2	3	4

For the following questions please circle the number between 1 and 7 that best applies to you

29. How would you rate your overall health during the past week?

1 2 3 4 5 6 7

Very poor Excellent

30. How would you rate your overall quality of life during the past week?

1 2 3 4 5 6 7

Very poor Excellent

© Copyright 1995 EORTC Quality of Life Group. All rights reserved. Version 3.0

Printed copy from PhlexEview - Property of Eisai.

EORTC QLQ – HCC18

Patients sometimes report that they have the following symptoms or problems. Please indicate the extent to which you have experienced these symptoms or problems during the past week. Please answer by circling the number that best applies to you.

During the past week:	Not at all	A little	Quite a bit	Very much
31. Did you feel thirsty?	1	2	3	4
32. Have you had problems with your sense of taste?	1	2	3	4
33. Have you lost muscle from your arms or legs?	1	2	3	4
34. Have you had abdominal swelling?	1	2	3	4
35. Have you been concerned by the appearance of your abdomen?	1	2	3	4
36. Have you been concerned by your skin or eyes being yellow (jaundiced)?	1	2	3	4
37. Have you had itching?	1	2	3	4
38. Have you had pain in your shoulder?	1	2	3	4
39. Have you had abdominal pain?	1	2	3	4
40. Have you had fevers?	1	2	3	4
41. Have you had chills?	1	2	3	4
42. Have you worried about getting enough nourishment?	1	2	3	4
43. Have you felt full up too quickly after beginning to eat?	1	2	3	4
44. Have you worried about your weight being too low?	1	2	3	4
45. Have you been less active than you would like to be?	1	2	3	4
46. Have you found it difficult to finish things?	1	2	3	4
47. Have you needed to sleep during the day?	1	2	3	4
**During the past four weeks:**				
48. Has the disease or treatment had any effect on your sex life?	1	2	3	4

© QLQ-HCC18 Copyright 2002 EORTC Study Group on Quality of life. All rights reserved.

Health Questionnaire

English version for the US

US (English) © 1998 EuroQol Group. EQ-5D™ is a trade mark of the EuroQol Group

By placing a checkmark in one box in each group below, please indicate which statements best describe your own health state today.

Mobility

I have no problems in walking about ❑

I have some problems in walking about ❑

I am confined to bed ❑

Self-Care

I have no problems with self-care ❑

I have some problems washing or dressing myself ❑

I am unable to wash or dress myself ❑

Usual Activities (*e.g. work, study, housework, family or leisure activities)*

I have no problems with performing my usual activities ❑

I have some problems with performing my usual activities ❑

I am unable to perform my usual activities ❑

Pain/Discomfort

I have no pain or discomfort ❑

I have moderate pain or discomfort ❑

I have extreme pain or discomfort ❑

Anxiety/Depression

I am not anxious or depressed ❑

I am moderately anxious or depressed ❑

I am extremely anxious or depressed ❑

US (English) © 1998 EuroQol Group. EQ-5D™ is a trade mark of the EuroQol Group

To help people say how good or bad a health state is, we have drawn a scale (rather like a thermometer) on which the best state you can imagine is marked 100 and the worst state you can imagine is marked 0.

We would like you to indicate on this scale how good or bad your own health is today, in your opinion. Please do this by drawing a line from the box below to whichever point on the scale indicates how good or bad your health state is today.

Your own health state today

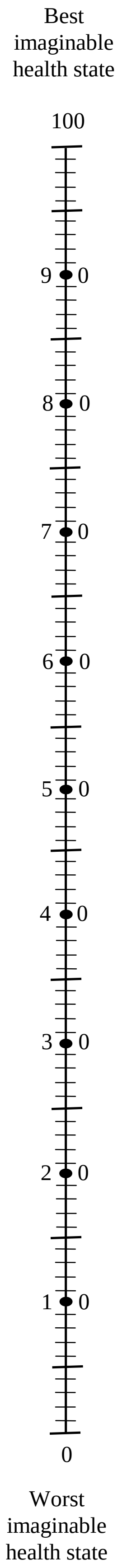

US (English) © 1998 EuroQol Group. EQ-5D™ is a trade mark of the EuroQol Group
